# Supplementary material for: Genetic Variants in the NOD-like Receptor Signaling Pathway Are Associated with HIV-1/AIDS in a Northern Chinese Population
Source: Int J Mol Sci. 2025 Apr 8;26(8):3484. doi: 10.3390/ijms26083484 (PMC12026778; doi:10.3390/ijms26083484)
Supplement: Supplementary file 1 [file ijms-26-03484-s001.zip › Supplementary_Table_S5_R3.docx]

**Table S5. Association between alleles of 37 candidate SNPs and CD4^+^ T lymphocyte count**

| Gene | SNP | Allele | Allele frequency in subgroup^a^ | | *p* value | OR (95%CI) |
| --- | --- | --- | --- | --- | --- | --- |
| CD4^+^ T cell counts (cells/μL) |  |  | < 200 | ≥ 200 |  |  |
| *CASP1* | *rs530537* | C | 56(0.226) | 161(0.214) | 0.698 | 1.017(0.758-1.512) |
|  |  | T | 192(0.774) | 591(0.786) |  | 1 (ref) |
| *STAT1* | *rs2066804* | G | 153(0.617) | 408(0.543) | **0.041** | 1.358(1.013-1.821) |
|  |  | A | 95(0.383) | 344(0.457) |  | 1 (ref) |
| *STAT1* | *rs1467199* | C | 135(0.544) | 363(0.483) | 0.092 | 1.280(0.960-1.707) |
|  |  | G | 113(0.456) | 389(0.517) |  | 1 (ref) |
| *OAS1* | *rs10774671* | G | 74(0.298) | 221(0.294) | 0.893 | 1.022(0.746-1.399) |
|  |  | A | 174(0.702) | 531(0.706) |  | 1 (ref) |
| *OAS1* | *rs1131454* | G | 119(0.480) | 357(0.475) | 0.889 | 1.021(0.766-1.360) |
|  |  | A | 129(0.520) | 395(0.525) |  | 1 (ref) |
| *IL18* | *rs549908* | G | 29(0.117) | 81(0.102) | 0.687 | 1.097(0.699-1.722) |
|  |  | T | 219(0.883) | 671(0.892) |  | 1 (ref) |
| *IL18* | *rs360719* | G | 29(0.117) | 82(0.109) | 0.741 | 1.079(0.688-1.692) |
|  |  | A | 219(0.883) | 668(0.891) |  | 1 (ref) |
| *IL18* | *rs1946518* | T | 121(0.488) | 349(0.464) | 0.515 | 1.100(0.825-1.466) |
|  |  | G | 127(0.512) | 403(0.536) |  | 1 (ref) |
| *GSDMD* | *rs11551202* | A | 36(0.145) | 93(0.124) | 0.389 | 1.200(0.792-1.816) |
|  |  | G | 212(0.855) | 657(0.876) |  | 1 (ref) |
| *GSDMD* | *rs1545536* | C | 149(0.601) | 412(0.549) | 0.157 | 1.235(0.922-1.653) |
|  |  | T | 99(0.399) | 338(0.451) |  | 1 (ref) |
| *GSDMD* | *rs7834318* | A | 149(0.601) | 426(0.566) | 0.343 | 1.152(0.860-1.543) |
|  |  | C | 99(0.399) | 326(0.434) |  | 1 (ref) |
| *NLRP3* | *rs10754558* | C | 139(0.560) | 408(0.543) | 0.623 | 1.075(0.805-1.435) |
|  |  | G | 109(0.440) | 344(0.457) |  | 1 (ref) |
| *NLRP3* | *rs4612666* | C | 144(0.581) | 409(0.544) | 0.313 | 1.161(0.869-1.552) |
|  |  | T | 104(0.419) | 343(0.456) |  | 1 (ref) |
| *NLRP3* | *rs3806265* | T | 134(0.540) | 401(0.533) | 0.846 | 1.029(0.771-1.372) |
|  |  | C | 114(0.460) | 351(0.467) |  | 1 (ref) |
| *NLRP3* | *rs1539019* | C | 146(0.589) | 396(0.527) | 0.089 | 1.287(0.962-1.721) |
|  |  | A | 102(0.411) | 356(0.473) |  | 1 (ref) |
| *IL1B* | *rs4848306* | G | 126(0.508) | 376(0.501) | 0.854 | 1.027(0.771-1.369) |
|  |  | A | 122(0.492) | 374(0.499) |  | 1 (ref) |
| *IL1B* | *rs3136558* | A | 156(0.629) | 416(0.590) | 0.281 | 1.178(0.874-1.587) |
|  |  | G | 92(0.371) | 289(0.410) |  | 1 (ref) |
| *IL1B* | *rs2853550* | G | 226(0.911) | 680(0.904) | 0.742 | 1.088(0.659-1.794) |
|  |  | A | 22(0.089) | 72(0.096) |  | 1 (ref) |
| *IL1B* | *rs16944* | A | 118(0.476) | 350(0.465) | 0.776 | 1.043(0.782-1.390) |
|  |  | G | 130(0.524) | 402(0.535) |  | 1 (ref) |
| *IL1B* | *rs1143623* | C | 156(0.629) | 449(0.597) | 0.372 | 1.144(0.851-1.538) |
|  |  | G | 92(0.371) | 303(0.403) |  | 1 (ref) |
| *MAVS* | *rs7262903* | A | 33(0.133) | 90(0.120) | 0.578 | 1.129(0.736-1.731) |
|  |  | C | 215(0.867) | 662(0.880) |  | 1 (ref) |
| *MAVS* | *rs17857295* | G | 123(0.496) | 363(0.484) | 0.744 | 1.049(0.787-1.389) |
|  |  | C | 125(0.504) | 387(0.516) |  | 1 (ref) |
| *MAVS* | *rs6084497* | T | 98(0.395) | 284(0.378) | 0.623 | 1.077(0.802-1.445) |
|  |  | C | 150(0.605) | 468(0.622) |  | 1 (ref) |
| *MAVS* | *rs16989000* | A | 145(0.585) | 438(0.582) | 0.951 | 1.009(0.754-1.350) |
|  |  | C | 103(0.415) | 314(0.418) |  | 1 (ref) |
| *MAVS* | *rs6515831* | T | 190(0.766) | 575(0.765) | 0.961 | 1.008(0.719-1.415) |
|  |  | C | 58(0.234) | 177(0.235) |  | 1 (ref) |
| *MAVS* | *rs57173648* | T | 17(0.069) | 47(0.063) | 0.736 | 1.104(0.622-1.960) |
|  |  | C | 231(0.931) | 705(0.938) |  | 1 (ref) |
| *MAVS* | *rs867335* | T | 186(0.750) | 551(0.735) | 0.634 | 1.083(0.779-1.507) |
|  |  | A | 62(0.250) | 199(0.265) |  | 1 (ref) |
| *JAK1* | *rs7531799* | T | 125(0.504) | 348(0.463) | 0.259 | 1.180(0.885-1.572) |
|  |  | C | 123(0.496) | 404(0.537) |  | 1 (ref) |
| *JAK1* | *rs4244165* | T | 86(0.347) | 253(0.337) | 0.786 | 1.043(0.771-1.411) |
|  |  | G | 162(0.653) | 497(0.663) |  | 1 (ref) |
| *JAK1* | *rs1039125* | T | 150(0.605) | 437(0.581) | 0.511 | 1.103(0.823-1.479) |
|  |  | C | 98(0.395) | 315(0.419) |  | 1 (ref) |
| *JAK1* | *rs56818621* | C | 160(0.645) | 461(0.615) | 0.391 | 1.140(0.845-1.537) |
|  |  | G | 88(0.355) | 289(0.385) |  | 1 (ref) |
| *JAK1* | *rs11579758* | G | 161(0.649) | 481(0.640) | 0.785 | 1.043(0.772-1.408) |
|  |  | A | 87(0.351) | 271(0.360) |  | 1 (ref) |
| *JAK1* | *rs567354* | A | 106(0.427) | 300(0.399) | 0.428 | 1.125(0.841-1.504) |
|  |  | G | 142(0.573) | 452(0.601) |  | 1 (ref) |
| *JAK1* | *rs490178* | G | 72(0.290) | 202(0.269) | 0.521 | 1.110(0.807-1.525) |
|  |  | A | 176(0.710) | 548(0.731) |  | 1 (ref) |
| *JAK1* | *rs705509* | A | 112(0.452) | 318(0.424) | 0.447 | 1.119(0.838-1.494) |
|  |  | G | 136(0.548) | 432(0.576) |  | 1 (ref) |
| *JAK1* | *rs489500* | C | 168(0.677) | 506(0.675) | 0.936 | 1.013(0.745-1.376) |
|  |  | G | 80(0.323) | 244(0.325) |  | 1 (ref) |
| *JAK1* | *rs310241* | G | 71(0.286) | 191(0.255) | 0.327 | 1.174(0.852-0.618) |
|  |  | A | 177(0.714) | 559(0.745) |  | 1 (ref) |
| CD4^+^ T cell counts (cells/μL) |  |  | <500 | ≥500 |  |  |
| *CASP1* | *rs530537* | C | 167(0.223) | 50(0.198) | 0.408 | 1.161(0.815-1.655) |
|  |  | T | 581(0.777) | 202(0.802) |  | 1 (ref) |
| *STAT1* | *rs2066804* | G | 437(0.584) | 124(0.492) | **0.011** | 1.450(1.089-1.932) |
|  |  | A | 311(0.416) | 128(0.508) |  | 1 (ref) |
| *STAT1* | *rs1467199* | C | 385(0.515) | 113(0.448) | 0.069 | 1.305(0.797-1.738) |
|  |  | G | 363(0.485) | 139(0.552) |  | 1 (ref) |
| *OAS1* | *rs10774671* | A | 531(0.710) | 174(0.690) | 0.559 | 1.097(0.804-1.496) |
|  |  | G | 217(0.290) | 78(0.310) |  | 1 (ref) |
| *OAS1* | *rs1131454* | A | 396(0.529) | 128(0.508) | 0.555 | 1.090(0.819-1.450) |
|  |  | G | 352(0.471) | 124(0.492) |  | 1 (ref) |
| *IL18* | *rs549908* | G | 85(0.114) | 25(0.099) | 0.527 | 1.164(0.727-1.864) |
|  |  | T | 663(0.886) | 227(0.901) |  | 1 (ref) |
| *IL18* | *rs360719* | G | 85(0.114) | 26(0.103) | 0.638 | 1.118(0.702-1.779) |
|  |  | A | 661(0.886) | 226(0.897) |  | 1 (ref) |
| *IL18* | *rs1946518* | T | 352(0.471) | 118(0.468) | 0.949 | 1.009(0.758-1.344) |
|  |  | G | 396(0.529) | 134(0.532) |  | 1 (ref) |
| *GSDMD* | *rs11551202* | A | 98(0.131) | 31(0.123) | 0.733 | 1.078(0.700-1.660) |
|  |  | G | 648(0.869) | 221(0.877) |  | 1 (ref) |
| *GSDMD* | *rs1545536* | C | 424(0.568) | 137(0.544) | 0.494 | 1.105(0.829-1.473) |
|  |  | T | 322(0.432) | 115(0.456) |  | 1 (ref) |
| *GSDMD* | *rs7834318* | A | 432(0.578) | 143(0.567) | 0.78 | 1.042(0.781-1.390) |
|  |  | C | 316(0.422) | 109(0.433) |  | 1 (ref) |
| *NLRP3* | *rs10754558* | C | 413(0.539) | 134(0.532) | 0.838 | 1.030(0.775-1.370) |
|  |  | G | 353(0.461) | 118(0.468) |  | 1 (ref) |
| *NLRP3* | *rs4612666* | T | 337(0.451) | 110(0.437) | 0.699 | 1.058(0.794-1.411) |
|  |  | C | 411(0.549) | 142(0.563) |  | 1 (ref) |
| *NLRP3* | *rs3806265* | C | 353(0.472) | 112(0.444) | 0.449 | 1.117(0.838-1.488) |
|  |  | T | 395(0.528) | 140(0.556) |  | 1 (ref) |
| *NLRP3* | *rs1539019* | C | 412(0.551) | 130(0.516) | 0.336 | 1.151(0.865-1.532) |
|  |  | A | 336(0.449) | 122(0.484) |  | 1 (ref) |
| *IL1B* | *rs4848306* | A | 383(0.513) | 113(0.448) | 0.074 | 1.298(0.974-1.729) |
|  |  | G | 363(0.487) | 139(0.552) |  | 1 (ref) |
| *IL1B* | *rs3136558* | A | 466(0.625) | 151(0.599) | 0.472 | 1.113(0.831-1.491) |
|  |  | G | 280(0.375) | 101(0.401) |  | 1 (ref) |
| *IL1B* | *rs2853550* | A | 76(0.102) | 18(0.071) | 0.156 | 1.470(0.861-2.510) |
|  |  | G | 672(0.898) | 234(0.929) |  | 1 (ref) |
| *IL1B* | *rs16944* | G | 411(0.549) | 121(0.480) | 0.057 | 1.320(0.992-1.758) |
|  |  | A | 337(0.451) | 131(0.520) |  | 1 (ref) |
| *IL1B* | *rs1143623* | C | 470(0.628) | 135(0.536) | **0.009** | 1.465(1.098-1.955) |
|  |  | G | 278(0.372) | 117(0.464) |  | 1 (ref) |
| *MAVS* | *rs7262903* | A | 98(0.131) | 25(0.099) | 0.184 | 1.369(0.860-2.178) |
|  |  | C | 650(0.869) | 227(0.901) |  | 1 (ref) |
| *MAVS* | *rs17857295* | G | 372(0.499) | 114(0.452) | 0.204 | 1.204(0.904-1.604) |
|  |  | C | 374(0.501) | 138(0.548) |  | 1 (ref) |
| *MAVS* | *rs6084497* | C | 474(0.634) | 144(0.571) | 0.079 | 1.297(0.970-1.735) |
|  |  | T | 274(0.366) | 108(0.429) |  | 1 (ref) |
| *MAVS* | *rs16989000* | A | 438(0.586) | 145(0.575) | 0.777 | 1.043(0.781-1.392) |
|  |  | C | 310(0.414) | 107(0.425) |  | 1 (ref) |
| *MAVS* | *rs6515831* | T | 579(0.774) | 186(0.738) | 0.244 | 1.216(0.875-1.689) |
|  |  | C | 169(0.226) | 66(0.262) |  | 1 (ref) |
| *MAVS* | *rs57173648* | C | 704(0.941) | 232(0.921) | 0.249 | 1.379(0.797-2.388) |
|  |  | T | 44(0.059) | 20(0.079) |  | 1 (ref) |
| *MAVS* | *rs867335* | T | 557(0.747) | 180(0.714) | 0.312 | 1.179(0.857-1.622) |
|  |  | A | 189(0.253) | 72(0.286) |  | 1 (ref) |
| *JAK1* | *rs7531799* | C | 403(0.539) | 124(0.492) | 0.199 | 1.206(0.906-1.605) |
|  |  | T | 345(0.461) | 128(0.508) |  | 1 (ref) |
| *JAK1* | *rs4244165* | T | 256(0.343) | 83(0.329) | 0.689 | 1.064(0.786-1.440) |
|  |  | G | 490(0.657) | 169(0.671) |  | 1 (ref) |
| *JAK1* | *rs1039125* | C | 315(0.421) | 98(0.389) | 0.369 | 1.143(0.854-1.531) |
|  |  | T | 433(0.579) | 154(0.611) |  | 1 (ref) |
| *JAK1* | *rs56818621* | G | 286(0.383) | 91(0.361) | 0.528 | 1.100(0.818-1.480) |
|  |  | C | 460(0.617) | 161(0.639) |  | 1 (ref) |
| *JAK1* | *rs11579758* | A | 269(0.360) | 89(0.353) | 0.853 | 1.029(0.763-1.386) |
|  |  | G | 479(0.640) | 163(0.647) |  | 1 (ref) |
| *JAK1* | *rs567354* | G | 452(0.604) | 142(0.563) | 0.254 | 1.183(0.886-1.579) |
|  |  | A | 296(0.396) | 110(0.437) |  | 1 (ref) |
| *JAK1* | *rs490178* | A | 546(0.732) | 178(0.706) | 0.432 | 1.135(0.828-1.556) |
|  |  | G | 200(0.268) | 74(0.294) |  | 1 (ref) |
| *JAK1* | *rs705509* | A | 325(0.436) | 105(0.417) | 0.599 | 1.081(0.809-1.443) |
|  |  | G | 421(0.564) | 147(0.583) |  | 1 (ref) |
| *JAK1* | *rs489500* | C | 511(0.685) | 163(0.647) | 0.263 | 1.187(0.879-1.604) |
|  |  | G | 235(0.315) | 89(0.353) |  | 1 (ref) |
| *JAK1* | *rs310241* | G | 196(0.263) | 66(0.262) | 0.979 | 1.004(0.726-1.390) |
|  |  | A | 550(0.737) | 186(0.738) |  | 1 (ref) |
| ^a^Results are shown as n (frequency). | | | | | | |
| Bold type indicates statistical significance (*p* < 0.05). | | | | | | |
