# Supplementary material for: Genetic Variants in the NOD-like Receptor Signaling Pathway Are Associated with HIV-1/AIDS in a Northern Chinese Population
Source: Int J Mol Sci. 2025 Apr 8;26(8):3484. doi: 10.3390/ijms26083484 (PMC12026778; doi:10.3390/ijms26083484)
Supplement: Supplementary file 1 [file ijms-26-03484-s001.zip › Supplementary_Table_S6_R3.docx]

**Table S6. Association between genotypes of 37 candidate SNPs and CD4^+^ T lymphocyte count**

| Gene | SNP | Genetic models | Genotype | Wilcoxon rank sum test *p* value |
| --- | --- | --- | --- | --- |
| *CASP1* | *rs530537* | dominant | CC+TC vs. TT | 0.672 |
|  |  | recessive | CC vs. TC+TT | 0.200 |
|  |  | codominant | CC vs. TT | 0.211 |
|  |  | codominant | TC vs. TT | 0.982 |
| *STAT1* | *rs2066804* | dominant | GG+GA vs. AA | **0.031** |
|  |  | recessive | GG vs. GA+AA | **0.004** |
|  |  | codominant | GG vs. AA | **0.003** |
|  |  | codominant | GA vs. AA | 0.176 |
| *STAT1* | *rs1467199* | dominant | CC+GC vs. GG | **0.036** |
|  |  | recessive | CC vs. GC+GG | 0.427 |
|  |  | codominant | CC vs. GG | 0.780 |
|  |  | codominant | GC vs. GG | 0.052 |
| *OAS1* | *rs10774671* | dominant | GG+GA vs. AA | 0.903 |
|  |  | recessive | GG vs. GA+AA | 0.836 |
|  |  | codominant | GG vs. AA | 0.913 |
|  |  | codominant | GA vs. AA | 0.850 |
| *OAS1* | *rs1131454* | dominant | GG+GA vs. AA | 0.484 |
|  |  | recessive | GG vs. GA+AA | 0.532 |
|  |  | codominant | GG vs. AA | 0.996 |
|  |  | codominant | GA vs. AA | 0.319 |
| *IL18* | *rs549908* | dominant | GG+TG vs. TT | 0.708 |
|  |  | recessive | GG vs. TG+TT | 0.526 |
|  |  | codominant | GG vs. TT | 0.515 |
|  |  | codominant | TG vs. TT | 0.778 |
| *IL18* | *rs360719* | dominant | GG+GA vs. AA | 0.763 |
|  |  | recessive | GG vs. GA+AA | 0.549 |
|  |  | codominant | GG vs. AA | 0.536 |
|  |  | codominant | GA vs. AA | 0.818 |
| *IL18* | *rs1946518* | dominant | TT+TG vs. GG | 0.550 |
|  |  | recessive | TT vs. TG+GG | 0.658 |
|  |  | codominant | TT vs. GG | 0.965 |
|  |  | codominant | TG vs. GG | 0.437 |
| *GSDMD* | *rs11551202* | dominant | AA+GA vs. GG | 0.412 |
|  |  | recessive | AA vs. GA+GG | 0.562 |
|  |  | codominant | AA vs. GG | 0.611 |
|  |  | codominant | GA vs. GG | 0.315 |
| *GSDMD* | *rs1545536* | dominant | CC+TC vs. TT | 0.371 |
|  |  | recessive | CC vs. TC+TT | 0.292 |
|  |  | codominant | CC vs. TT | 0.289 |
|  |  | codominant | TC vs. TT | 0.504 |
| *GSDMD* | *rs7834318* | dominant | AA+AC vs. CC | 0.285 |
|  |  | recessive | AA vs. AC+CC | 0.471 |
|  |  | codominant | AA vs. CC | 0.263 |
|  |  | codominant | AC vs. CC | 0.374 |
| *NLRP3* | *rs10754558* | dominant | CC+GC vs. GG | 0.783 |
|  |  | recessive | CC vs. GC+GG | 0.291 |
|  |  | codominant | CC vs. GG | 0.437 |
|  |  | codominant | GC vs. GG | 0.886 |
| *NLRP3* | *rs4612666* | dominant | CC+TC vs. TT | 0.518 |
|  |  | recessive | CC vs. TC+TT | 0.574 |
|  |  | codominant | CC vs. TT | 0.412 |
|  |  | codominant | TC vs. TT | 0.639 |
| *NLRP3* | *rs3806265* | dominant | TT+TC vs. CC | 0.692 |
|  |  | recessive | TT vs. TC+CC | 0.911 |
|  |  | codominant | TT vs. CC | 0.805 |
|  |  | codominant | TC vs. CC | 0.669 |
| *NLRP3* | *rs1539019* | dominant | CC+AC vs. AA | 0.113 |
|  |  | recessive | CC vs. AC+AA | 0.248 |
|  |  | codominant | CC vs. AA | 0.082 |
|  |  | codominant | AC vs. AA | 0.215 |
| *IL1B* | *rs4848306* | dominant | GG+GA vs. AA | 0.518 |
|  |  | recessive | GG vs. GA+AA | 0.333 |
|  |  | codominant | GG vs. AA | 0.290 |
|  |  | codominant | GA vs. AA | 0.752 |
| *IL1B* | *rs3136558* | dominant | AA+GA vs. GG | 0.764 |
|  |  | recessive | AA vs. GA+GG | 0.335 |
|  |  | codominant | AA vs. GG | 0.901 |
|  |  | codominant | GA vs. GG | 0.553 |
| *IL1B* | *rs2853550* | dominant | GG+GA vs. AA | 0.094 |
|  |  | recessive | GG vs. GA+AA | 0.510 |
|  |  | codominant | GG vs. AA | 0.097 |
|  |  | codominant | GA vs. AA | 0.091 |
| *IL1B* | *rs16944* | dominant | AA+GA vs. GG | 0.198 |
|  |  | recessive | AA vs. GA+GG | 0.670 |
|  |  | codominant | AA vs. GG | 0.329 |
|  |  | codominant | GA vs. GG | 0.218 |
| *IL1B* | *rs1143623* | dominant | CC+GC vs. GG | 0.144 |
|  |  | recessive | CC vs. GC+GG | 0.064 |
|  |  | codominant | CC vs. GG | 0.060 |
|  |  | codominant | GC vs. GG | 0.336 |
| *MAVS* | *rs7262903* | dominant | AA+AC vs. CC | 0.898 |
|  |  | recessive | AA vs. AC+CC | 0.137 |
|  |  | codominant | AA vs. CC | 0.135 |
|  |  | codominant | AC vs. CC | 0.838 |
| *MAVS* | *rs17857295* | dominant | GG+GC vs. CC | 0.375 |
|  |  | recessive | GG vs. GC+CC | 0.171 |
|  |  | codominant | GG vs. CC | 0.185 |
|  |  | codominant | GC vs. CC | 0.594 |
| *MAVS* | *rs6084497* | dominant | TT+TC vs. CC | 0.476 |
|  |  | recessive | TT vs. TC+CC | 0.388 |
|  |  | codominant | TT vs. CC | 0.334 |
|  |  | codominant | TC vs. CC | 0.640 |
| *MAVS* | *rs16989000* | dominant | AA+CA vs. CC | 0.747 |
|  |  | recessive | AA vs. CA+CC | 0.294 |
|  |  | codominant | AA vs. CC | 0.816 |
|  |  | codominant | CA vs. CC | 0.505 |
| *MAVS* | *rs6515831* | dominant | TT+TC vs. CC | 0.337 |
|  |  | recessive | TT vs. TC+CC | 0.518 |
|  |  | codominant | TT vs. CC | 0.296 |
|  |  | codominant | TC vs. CC | 0.432 |
| *MAVS* | *rs57173648* | dominant | TT+TC vs. CC | 0.548 |
|  |  | recessive | TT vs. TC+CC | 0.397 |
|  |  | codominant | TT vs. CC | 0.414 |
|  |  | codominant | TC vs. CC | 0.452 |
| *MAVS* | *rs867335* | dominant | TT+AT vs. AA | 0.537 |
|  |  | recessive | TT vs. AT+AA | 0.214 |
|  |  | codominant | TT vs. AA | 0.735 |
|  |  | codominant | AT vs. AA | 0.350 |
| *JAK1* | *rs7531799* | dominant | TT+TC vs. CC | 0.537 |
|  |  | recessive | TT vs. TC+CC | 0.615 |
|  |  | codominant | TT vs. CC | 0.495 |
|  |  | codominant | TC vs. CC | 0.638 |
| *JAK1* | *rs4244165* | dominant | TT+TG vs. GG | 0.665 |
|  |  | recessive | TT vs. TG+GG | 0.352 |
|  |  | codominant | TT vs. GG | 0.500 |
|  |  | codominant | TG vs. GG | 0.454 |
| *JAK1* | *rs1039125* | dominant | TT+TC vs. CC | 0.205 |
|  |  | recessive | TT vs. TC+CC | 0.711 |
|  |  | codominant | TT vs. CC | 0.275 |
|  |  | codominant | TC vs. CC | 0.218 |
| *JAK1* | *rs56818621* | dominant | CC+GC vs. GG | 0.177 |
|  |  | recessive | CC vs. GC+GG | 0.930 |
|  |  | codominant | CC vs. GG | 0.297 |
|  |  | codominant | GC vs. GG | 0.147 |
| *JAK1* | *rs11579758* | dominant | GG+GA vs. AA | 0.528 |
|  |  | recessive | GG vs. GA+AA | 0.370 |
|  |  | codominant | GG vs. AA | 0.828 |
|  |  | codominant | GA vs. AA | 0.355 |
| *JAK1* | *rs567354* | dominant | AA+GA vs. GG | 0.854 |
|  |  | recessive | AA vs. GA+GG | 0.705 |
|  |  | codominant | AA vs. GG | 0.711 |
|  |  | codominant | GA vs. GG | 0.938 |
| *JAK1* | *rs490178* | dominant | GG+GA vs. AA | 0.395 |
|  |  | recessive | GG vs. GA+AA | 0.934 |
|  |  | codominant | GG vs. AA | 0.817 |
|  |  | codominant | GA vs. AA | 0.382 |
| *JAK1* | *rs705509* | dominant | AA+GA vs. GG | 0.818 |
|  |  | recessive | AA vs. GA+GG | 0.142 |
|  |  | codominant | AA vs. GG | 0.331 |
|  |  | codominant | GA vs. GG | 0.438 |
| *JAK1* | *rs489500* | dominant | CC+GC vs. GG | 0.427 |
|  |  | recessive | CC vs. GC+GG | 0.663 |
|  |  | codominant | CC vs. GG | 0.398 |
|  |  | codominant | GC vs. GG | 0.509 |
| *JAK1* | *rs310241* | dominant | GG+GA vs. AA | 0.627 |
|  |  | recessive | GG vs. GA+AA | 0.863 |
|  |  | codominant | GG vs. AA | 0.946 |
|  |  | codominant | GA vs. AA | 0.564 |
| Bold type indicates statistical significance (*p* < 0.05). | | | | |
