# Supplementary material for: Genetic Variants in the NOD-like Receptor Signaling Pathway Are Associated with HIV-1/AIDS in a Northern Chinese Population
Source: Int J Mol Sci. 2025 Apr 8;26(8):3484. doi: 10.3390/ijms26083484 (PMC12026778; doi:10.3390/ijms26083484)
Supplement: Supplementary file 1 [file ijms-26-03484-s001.zip › Supplementary_Table_S7_R3.docx]

**Table S7. Association between alleles of 37 candidate SNPs and AIDS phase**

| Gene | SNP | Allele | Clinical phase | | *p* value | OR (95%CI) |
| --- | --- | --- | --- | --- | --- | --- |
|  |  |  | Ⅲ+Ⅳ^a^ | Ⅰ+Ⅱ^a^ |  |  |
| *CASP1* | *rs530537* | C | 113(0.226) | 104(0.208) | 0.490 | 1.112(0.823-1.502) |
|  |  | T | 387(0.774) | 396(0.792) |  | 1 (ref) |
| *STAT1* | *rs2066804* | G | 292(0.584) | 269(0.538) | 0.143 | 1.206(0.939-1.548) |
|  |  | A | 208(0.416) | 231(0.462) |  | 1 (ref) |
| *STAT1* | *rs1467199* | C | 255(0.510) | 243(0.486) | 0.448 | 1.101(0.859-1.411) |
|  |  | G | 245(0.490) | 257(0.514) |  | 1 (ref) |
| *OAS1* | *rs10774671* | G | 149(0.298) | 146(0.292) | 0.835 | 1.029(0.784-1.351) |
|  |  | A | 351(0.702) | 354(0.708) |  | 1 (ref) |
| *OAS1* | *rs1131454* | G | 247(0.494) | 229(0.458) | 0.254 | 1.155(0.901-1.481) |
|  |  | A | 253(0.506) | 271(0.542) |  | 1 (ref) |
| *IL18* | *rs549908* | G | 58(0.116) | 52(0.104) | 0.544 | 1.131(0.760-1.681) |
|  |  | T | 442(0.884) | 448(0.896) |  | 1 (ref) |
| *IL18* | *rs360719* | G | 58(0.116) | 53(0.106) | 0.599 | 1.112(0.749-1.650) |
|  |  | A | 440(0.884) | 447(0.894) |  | 1 (ref) |
| *IL18* | *rs1946518* | G | 244(0.488) | 226(0.452) | 0.254 | 1.156(0.901-1.482) |
|  |  | T | 256(0.512) | 274(0.548) |  | 1 (ref) |
| *GSDMD* | *rs11551202* | A | 434(0.871) | 435(0.870) | 0.944 | 1.013(0.700-1.467) |
|  |  | G | 64(0.129) | 65(0.130) |  | 1 (ref) |
| *GSDMD* | *rs1545536* | C | 282(0.566) | 279(0.558) | 0.792 | 1.034(0.805-1.328) |
|  |  | T | 216(0.434) | 221(0.442) |  | 1 (ref) |
| *GSDMD* | *rs7834318* | A | 290(0.580) | 285(0.570) | 0.749 | 1.042(0.811-1.339) |
|  |  | C | 210(0.420) | 215(0.430) |  | 1 (ref) |
| *NLRP3* | *rs10754558* | C | 287(0.574) | 260(0.520) | 0.086 | 1.244(0.969-1.596) |
|  |  | G | 213(0.426) | 240(0.480) |  | 1 (ref) |
| *NLRP3* | *rs4612666* | T | 224(0.448) | 223(0.446) | 0.949 | 1.008(0.786-1.294) |
|  |  | C | 276(0.552) | 277(0.554) |  | 1 (ref) |
| *NLRP3* | *rs3806265* | C | 237(0.474) | 228(0.456) | 0.568 | 1.075(0.838-1.378) |
|  |  | T | 263(0.526) | 272(0.544) |  | 1 (ref) |
| *NLRP3* | *rs1539019* | C | 281(0.562) | 261(0.522) | 0.204 | 1.175(0.916-1.507) |
|  |  | A | 219(0.438) | 239(0.478) |  | 1 (ref) |
| *IL1B* | *rs4848306* | A | 250(0.502) | 246(0.492) | 0.752 | 1.041(0.812-1.334) |
|  |  | G | 248(0.498) | 254(0.508) |  | 1 (ref) |
| *IL1B* | *rs3136558* | A | 308(0.618) | 309(0.618) | 0.988 | 1.002(0.776-1.294) |
|  |  | G | 190(0.382) | 191(0.382) |  | 1 (ref) |
| *IL1B* | *rs2853550* | A | 47(0.094) | 47(0.094) | 1.000 | 1.000(0.654-1.529) |
|  |  | G | 453(0.906) | 453(0.906) |  | 1 (ref) |
| *IL1B* | *rs16944* | G | 271(0.542) | 261(0.522) | 0.526 | 1.084(0.845-1.389) |
|  |  | A | 229(0.458) | 239(0.478) |  | 1 (ref) |
| *IL1B* | *rs1143623* | C | 307(0.614) | 298(0.596) | 0.560 | 1.078(0.837-1.390) |
|  |  | G | 193(0.386) | 202(0.404) |  | 1 (ref) |
| *MAVS* | *rs7262903* | C | 445(0.890) | 432(0.864) | 0.211 | 1.274(0.872-1.861) |
|  |  | A | 55(0.110) | 68(0.136) |  | 1 (ref) |
| *MAVS* | *rs17857295* | C | 251(0.504) | 235(0.470) | 0.282 | 1.146(0.894-1.469) |
|  |  | G | 247(0.496) | 265(0.530) |  | 1 (ref) |
| *MAVS* | *rs6084497* | C | 319(0.638) | 299(0.598) | 0.193 | 1.195(0.918-1.530) |
|  |  | T | 181(0.362) | 201(0.402) |  | 1 (ref) |
| *MAVS* | *rs16989000* | A | 303(0.606) | 280(0.560) | 0.140 | 1.208(0.940-1.554) |
|  |  | C | 197(0.394) | 220(0.440) |  | 1 (ref) |
| *MAVS* | *rs6515831* | C | 122(0.244) | 113(0.226) | 0.502 | 1.105(0.825-1.481) |
|  |  | T | 378(0.756) | 287(0.774) |  | 1 (ref) |
| *MAVS* | *rs57173648* | C | 468(0.936) | 468(0.936) | 1.000 | 1.000(0.603-1.659) |
|  |  | T | 32(0.064) | 32(0.064) |  | 1 (ref) |
| *MAVS* | *rs867335* | T | 372(0.747) | 365(0.730) | 0.541 | 1.092(0.823-1.448) |
|  |  | A | 126(0.253) | 135(0.270) |  | 1 (ref) |
| *JAK1* | *rs7531799* | T | 237(0.474) | 236(0.472) | 0.949 | 1.008(0.786-1.292) |
|  |  | C | 263(0.526) | 264(0.528) |  | 1 (ref) |
| *JAK1* | *rs4244165* | T | 171(0.343) | 168(0.336) | 0.806 | 1.033(0.795-1.343) |
|  |  | G | 327(0.657) | 332(0.664) |  | 1 (ref) |
| *JAK1* | *rs1039125* | C | 211(0.422) | 202(0.404) | 0.563 | 1.007(0.837-1.386) |
|  |  | T | 289(0.578) | 298(0.596) |  | 1 (ref) |
| *JAK1* | *rs56818621* | G | 194(0.390) | 183(0.366) | 0.443 | 1.105(0.856-1.428) |
|  |  | C | 304(0.610) | 317(0.634) |  | 1 (ref) |
| *JAK1* | *rs11579758* | A | 323(0.646) | 319(0.638) | 0.792 | 1.035(0.800-1.341) |
|  |  | G | 177(0.354) | 181(0.362) |  | 1 (ref) |
| *JAK1* | *rs567354* | G | 301(0.602) | 293(0.586) | 0.606 | 1.069(0.830-1.375) |
|  |  | A | 199(0.398) | 207(0.414) |  | 1 (ref) |
| *JAK1* | *rs490178* | G | 139(0.279) | 135(0.270) | 0.747 | 1.047(0.793-1.382) |
|  |  | A | 359(0.721) | 365(0.730) |  | 1 (ref) |
| *JAK1* | *rs705509* | A | 220(0.442) | 210(0.420) | 0.487 | 1.093(0.851-1.404) |
|  |  | G | 278(0.558) | 290(0.580) |  | 1 (ref) |
| *JAK1* | *rs489500* | C | 341(0.685) | 333(0.666) | 0.527 | 1.089(0.836-1.420) |
|  |  | G | 157(0.315) | 167(0.334) |  | 1 (ref) |
| *JAK1* | *rs310241* | A | 374(0.751) | 362(0.724) | 0.332 | 1.150(0.867-1.525) |
|  |  | G | 124(0.249) | 138(0.276) |  | 1 (ref) |
| ^a^Results are shown as n (frequency). | | | | | | |
| Bold type indicates statistical significance (*p* < 0.05). | | | | | | |
