# Supplementary material for: Genetic Variants in the NOD-like Receptor Signaling Pathway Are Associated with HIV-1/AIDS in a Northern Chinese Population
Source: Int J Mol Sci. 2025 Apr 8;26(8):3484. doi: 10.3390/ijms26083484 (PMC12026778; doi:10.3390/ijms26083484)
Supplement: Supplementary file 1 [file ijms-26-03484-s001.zip › Supplementary_Table_S8_R3.docx]

**Table S8. Association between genotypes of 37 candidate SNPs and AIDS phase**

| Gene | SNP | Genetic models | Genotype | Clinical stage | | *p* value | OR (95%CI) |
| --- | --- | --- | --- | --- | --- | --- | --- |
|  |  |  |  | Ⅲ+Ⅳ^a^ | Ⅰ+Ⅱ^a^ |  |  |
| *CASP1* | *rs530537* | dominant (CC+TC vs. TT) | CC+TC | 103(0.412) | 92(0.368) | 0.313 | 1.203(0.840-1.725) |
|  |  |  | TT | 147(0.588) | 158(0.632) |  | 1 (ref) |
|  |  | recessive (CC vs. TC+TT) | CC | 10(0.040) | 12(0.048) | 0.663 | 0.826(0.350-1.949) |
|  |  |  | TC+TT | 240(0.960) | 238(0.952) |  | 1 (ref) |
|  |  | codominant (CC vs. TT) | CC | 10(0.064) | 12(0.071) | 0.804 | 0.896(0.376-2.135) |
|  |  |  | TT | 147(0.936) | 158(0.929) |  | 1 (ref) |
|  |  | codominant (TC vs. TT) | TC | 93(0.388) | 80(0.336) | 0.243 | 1.249(0.860-1.816) |
|  |  |  | TT | 147(0.613) | 158(0.664) |  | 1 (ref) |
| *STAT1* | *rs2066804* | dominant (GG+GA vs. AA) | GG+GA | 210(0.840) | 200(0.800) | 0.244 | 1.313(0.830-2.076) |
|  |  |  | AA | 40(0.160) | 50(0.200) |  | 1 (ref) |
|  |  | recessive (GG vs. GA+AA) | GG | 82(0.328) | 69(0.276) | 0.205 | 1.280(0.873-1.878) |
|  |  |  | GA+AA | 168(0.672) | 181(0.724) |  | 1 (ref) |
|  |  | codominant (GG vs. AA) | GG | 82(0.672) | 69(0.580) | 0.139 | 1.486(0.879-2.511) |
|  |  |  | AA | 40(0.328) | 50(0.420) |  | 1 (ref) |
|  |  | codominant (GA vs. AA) | GA | 128(0.762) | 131(0.724) | 0.416 | 1.221(0.754-1.978) |
|  |  |  | AA | 40(0.238) | 50(0.276) |  | 1 (ref) |
| *STAT1* | *rs1467199* | dominant (CC+GC vs. GG) | CC+GC | 196(0.784) | 181(0.724) | 0.119 | 1.384(0.919-2.084) |
|  |  |  | GG | 54(0.216) | 69(0.276) |  | 1 (ref) |
|  |  | recessive (CC vs. GC+GG) | CC | 59(0.236) | 62(0.248) | 0.754 | 0.937(0.622-1.411) |
|  |  |  | GC+GG | 191(0.764) | 188(0.752) |  | 1 (ref) |
|  |  | codominant (CC vs. GG) | CC | 59(0.522) | 62(0.473) | 0.447 | 1.216(0.735-2.013) |
|  |  |  | GG | 54(0.478) | 69(0.527) |  | 1 (ref) |
|  |  | codominant (GC vs. GG) | GC | 137(0.717) | 119(0.633) | 0.080 | 1.471(0.954-2.267) |
|  |  |  | GG | 54(0.283) | 69(0.367) |  | 1 (ref) |
| *OAS1* | *rs10774671* | dominant (GG+GA vs. AA) | GG+GA | 128(0.512) | 117(0.468) | 0.325 | 1.193(0.840-1.694) |
|  |  |  | AA | 122(0.488) | 133(0.532) |  | 1 (ref) |
|  |  | recessive (GG vs. GA+AA) | GG | 21(0.084) | 29(0.116) | 0.233 | 0.699(0.387-1.262) |
|  |  |  | GA+AA | 229(0.916) | 221(0.884) |  | 1 (ref) |
|  |  | codominant (GG vs. AA) | GG | 21(0.147) | 29(0.179) | 0.449 | 0.789(0.428-1.457) |
|  |  |  | AA | 122(0.853) | 133(0.821) |  | 1 (ref) |
|  |  | codominant (GA vs. AA) | GA | 107(0.467) | 88(0.398) | 0.139 | 1.326(0.912-1.927) |
|  |  |  | AA | 122(0.533) | 133(0.602) |  | 1 (ref) |
| *OAS1* | *rs1131454* | dominant (GG+GA vs. AA) | GG+GA | 188(0.752) | 166(0.664) | **0.030** | 1.534(1.040-2.264) |
|  |  |  | AA | 62(0.248) | 84(0.336) |  | 1 (ref) |
|  |  | recessive (GG vs. GA+AA) | GG | 59(0.236) | 63(0.252) | 0.677 | 0.917(0.610-1.379) |
|  |  |  | GA+AA | 191(0.764) | 187(0.748) |  | 1 (ref) |
|  |  | codominant (GG vs. AA) | GG | 59(0.488) | 63(0.429) | 0.334 | 1.269(0.782-2.058) |
|  |  |  | AA | 62(0.512) | 84(0.571) |  | 1 (ref) |
|  |  | codominant (GA vs. AA) | GA | 129(0.675) | 103(0.551) | **0.013** | 1.697(1.117-2.577) |
|  |  |  | AA | 62(0.325) | 84(0.449) |  | 1 (ref) |
| *IL18* | *rs549908* | dominant (GG+TG vs. TT) | GG+TG | 56(0.224) | 51(0.204) | 0.586 | 1.126(0.734-1.728) |
|  |  |  | TT | 194(0.776) | 199(0.796) |  | 1 (ref) |
|  |  | recessive (GG vs. TG+TT) | GG | 2(0.008) | 1(0.004) | 1.000 | 2.008(0.181-22.288) |
|  |  |  | TG+TT | 248(0.992) | 249(0.996) |  | 1 (ref) |
|  |  | codominant (GG vs. TT) | GG | 2(0.010) | 1(0.005) | 0.620 | 2.052(0.185-22.810) |
|  |  |  | TT | 194(0.990) | 199(0.995) |  | 1 (ref) |
|  |  | codominant (TG vs. TT) | TG | 54(0.218) | 50(0.201) | 0.643 | 1.108(0.719-1.707) |
|  |  |  | TT | 194(0.782) | 199(0.799) |  | 1 (ref) |
| *IL18* | *rs360719* | dominant (GG+GA vs. AA) | GG+GA | 57(0.229) | 52(0.208) | 0.572 | 1.130(0.739-1.729) |
|  |  |  | AA | 192(0.771) | 198(0.792) |  | 1 (ref) |
|  |  | recessive (GG vs. GA+AA) | GG | 1(0.004) | 1(0.004) | 1.000 | 1.004(0.062-16.012) |
|  |  |  | GA+AA | 248(0.996) | 249(0.996) |  | 1 (ref) |
|  |  | codominant (GG vs. AA) | GG | 1(0.005) | 1(0.005) | 1.000 | 1.031(0.064-16.605) |
|  |  |  | AA | 192(0.995) | 198(0.995) |  | 1 (ref) |
|  |  | codominant (GA vs. AA) | GA | 56(0.226) | 51(0.205) | 0.569 | 1.132(0.738-1.738) |
|  |  |  | AA | 192(0.774) | 198(0.795) |  | 1 (ref) |
| *IL18* | *rs1946518* | dominant (GG+TG vs. TT) | GG+TG | 196(0.784) | 198(0.792) | 0.827 | 0.953(0.621-1.464) |
|  |  |  | TT | 54(0.216) | 52(0.208) |  | 1 (ref) |
|  |  | recessive (GG vs. TG+TT) | GG | 60(0.240) | 76(0.304) | 0.108 | 0.723(0.487-1.074) |
|  |  |  | TG+TT | 190(0.760) | 174(0.696) |  | 1 (ref) |
|  |  | codominant (GG vs. TT) | GG | 60(0.526) | 76(0.594) | 0.291 | 0.760(0.457-1.265) |
|  |  |  | TT | 54(0.474) | 52(0.406) |  | 1 (ref) |
|  |  | codominant (TG vs. TT) | TG | 136(0.716) | 122(0.701) | 0.759 | 1.073(0.683-1.688) |
|  |  |  | TT | 54(0.284) | 52(0.299) |  | 1 (ref) |
| *GSDMD* | *rs11551202* | dominant (AA+GA vs. GG) | AA+GA | 59(0.237) | 60(0.240) | 0.936 | 0.983(0.651-1.484) |
|  |  |  | GG | 190(0.763) | 190(0.760) |  | 1 (ref) |
|  |  | recessive (AA vs. GA+GG) | AA | 5(0.02) | 5(0.02) | 1.000 | 1.004(0.287-3.512) |
|  |  |  | GA+GG | 244(0.98) | 245(0.98) |  | 1 (ref) |
|  |  | codominant (AA vs. GG) | AA | 5(0.026) | 5(0.026) | 1.000 | 1.000(0.285-3.511) |
|  |  |  | GG | 190(0.974) | 190(0.974) |  | 1 (ref) |
|  |  | codominant (GA vs. GG) | GA | 54(0.221) | 55(0.224) | 0.933 | 0.982(0.641-1.503) |
|  |  |  | GG | 190(0.779) | 190(0.776) |  | 1 (ref) |
| *GSDMD* | *rs1545536* | dominant (CC+TC vs. TT) | CC+TC | 205(0.823) | 203(0.812) | 0.744 | 1.079(0.685-1.700) |
|  |  |  | TT | 44(0.177) | 47(0.188) |  | 1 (ref) |
|  |  | recessive (CC vs. TC+TT) | CC | 77(0.309) | 76(0.304) | 0.899 | 1.025(0.701-1.500) |
|  |  |  | TC+TT | 172(0.691) | 174(0.696) |  | 1 (ref) |
|  |  | codominant (CC vs. TT) | CC | 77(0.636) | 76(0.618) | 0.765 | 1.082(0.644-1.819) |
|  |  |  | TT | 44(0.364) | 47(0.382) |  | 1 (ref) |
|  |  | codominant (TC vs. TT) | TC | 128(0.744) | 127(0.730) | 0.763 | 1.077(0.667-1.738) |
|  |  |  | TT | 44(0.256) | 47(0.270) |  | 1 (ref) |
| *GSDMD* | *rs7834318* | dominant (AA+AC vs. CC) | AA+AC | 202(0.808) | 208(0.832) | 0.485 | 0.850(0.538-1.342) |
|  |  |  | CC | 48(0.892) | 42(0.168) |  | 1 (ref) |
|  |  | recessive (AA vs. AC+CC) | AA | 88(0.352) | 77(0.308) | 0.295 | 1.220(0.840-1.773) |
|  |  |  | AC+CC | 162(0.648) | 173(0.692) |  | 1 (ref) |
|  |  | codominant (AA vs. CC) | AA | 88(0.647) | 77(0.647) | 1.000 | 1.000(0.598-1.673) |
|  |  |  | CC | 48(0.353) | 42(0.353) |  | 1 (ref) |
|  |  | codominant (AC vs. CC) | AC | 114(0.704) | 131(0.757) | 0.269 | 0.761(0.469-1.236) |
|  |  |  | CC | 48(0.296) | 42(0.243) |  | 1 (ref) |
| *NLRP3* | *rs10754558* | dominant (CC+GC vs. GG) | CC+GC | 196(0.784) | 192(0.768) | 0.668 | 1.096(0.720-1.670) |
|  |  |  | GG | 54(0.216) | 58(0.232) |  | 1 (ref) |
|  |  | recessive (CC vs. GC+GG) | CC | 91(0.364) | 68(0.272) | **0.027** | 1.532(1.048-2.239) |
|  |  |  | GC+GG | 159(0.636) | 182(0.728) |  | 1 (ref) |
|  |  | codominant (CC vs. GG) | CC | 91(0.628) | 68(0.540) | 0.143 | 1.437(0.884-2.337) |
|  |  |  | GG | 54(0.372) | 58(0.460) |  | 1 (ref) |
|  |  | codominant (GC vs. GG) | GC | 105(0.660) | 124(0.681) | 0.681 | 0.909(0.578-1.430) |
|  |  |  | GG | 54(0.340) | 58(0.319) |  | 1 (ref) |
| *NLRP3* | *rs4612666* | dominant (TT+TC vs. CC) | TT+TC | 183(0.732) | 177(0.708) | 0.550 | 1.126(0.762-1.665) |
|  |  |  | CC | 67(0.268) | 73(0.292) |  | 1 (ref) |
|  |  | recessive (TT vs. TC+CC) | TT | 41(0.164) | 46(0.184) | 0.555 | 0.870(0.548-1.382) |
|  |  |  | TC+CC | 209(0.836) | 204(0.816) |  | 1 (ref) |
|  |  | codominant (TT vs. CC) | TT | 41(0.380) | 46(0.387) | 0.915 | 0.971(0.568-1.660) |
|  |  |  | CC | 67(0.620) | 73(0.613) |  | 1 (ref) |
|  |  | codominant (TC vs. CC) | TC | 142(0.679) | 131(0.642) | 0.424 | 1.181(0.785-1.776) |
|  |  |  | CC | 67(0.321) | 73(0.358) |  | 1 (ref) |
| *NLRP3* | *rs3806265* | dominant (CC+TC vs. TT) | CC+TC | 186(0.744) | 179(0.716) | 0.481 | 1.153(0.776-1.712) |
|  |  |  | TT | 64(0.256) | 71(0.284) |  | 1 (ref) |
|  |  | recessive (CC vs. TC+TT) | CC | 51(0.204) | 49(0.196) | 0.823 | 1.051(0.678-1.630) |
|  |  |  | TC+TT | 199(0.796) | 201(0.804) |  | 1 (ref) |
|  |  | codominant (CC vs. TT) | CC | 51(0.443) | 49(0.408) | 0.586 | 1.155(0.688-1.937) |
|  |  |  | TT | 64(0.557) | 71(0.592) |  | 1 (ref) |
|  |  | codominant (TC vs. TT) | TC | 135(0.678) | 130(0.647) | 0.504 | 1.152(0.761-1.744) |
|  |  |  | TT | 64(0.322) | 71(0.353) |  | 1 (ref) |
| *NLRP3* | *rs1539019* | dominant (CC+AC vs. AA) | CC+AC | 205(0.820) | 189(0.756) | 0.080 | 1.470(0.954-2.267) |
|  |  |  | AA | 45(0.180) | 61(0.244) |  | 1 (ref) |
|  |  | recessive (CC vs. AC+AA) | CC | 76(0.304) | 72(0.288) | 0.695 | 1.080(0.735-1.586) |
|  |  |  | AC+AA | 174(0.696) | 178(0.712) |  | 1 (ref) |
|  |  | codominant (CC vs. AA) | CC | 76(0.628) | 72(0.541) | 0.161 | 1.431(0.866-2.364) |
|  |  |  | AA | 45(0.372) | 61(0.459) |  | 1 (ref) |
|  |  | codominant (AC vs. AA) | AC | 129(0.741) | 117(0.657) | 0.086 | 1.495(0.944-2.366) |
|  |  |  | AA | 45(0.259) | 61(0.343) |  | 1 (ref) |
| *IL1B* | *rs4848306* | dominant (AA+GA vs. GG) | AA+GA | 187(0.751) | 189(0.756) | 0.897 | 0.973(0.648-1.463) |
|  |  |  | GG | 62(0.249) | 61(0.244) |  | 1 (ref) |
|  |  | recessive (AA vs. GA+GG) | AA | 63(0.253) | 57(0.228) | 0.513 | 1.147(0.760-1.730) |
|  |  |  | GA+GG | 186(0.747) | 193(0.772) |  | 1 (ref) |
|  |  | codominant (AA vs. GG) | AA | 63(0.504) | 57(0.483) | 0.744 | 1.087(0.657-1.799) |
|  |  |  | GG | 62(0.496) | 61(0.517) |  | 1 (ref) |
|  |  | codominant (GA vs. GG) | GA | 124(0.667) | 132(0.684) | 0.720 | 0.924(0.601-1.421) |
|  |  |  | GG | 62(0.333) | 61(0.316) |  | 1 (ref) |
| *IL1B* | *rs3136558* | dominant (GG+GA vs. AA) | GG+GA | 154(0.618) | 161(0.644) | 0.555 | 0.896(0.623-1.289) |
|  |  |  | AA | 95(0.382) | 89(0.356) |  | 1 (ref) |
|  |  | recessive (GG vs. GA+AA) | GG | 36(0.145) | 30(0.120) | 0.418 | 1.239(0.737-2.084) |
|  |  |  | GA+AA | 213(0.855) | 220(0.880) |  | 1 (ref) |
|  |  | codominant (GG vs. AA) | GG | 36(0.275) | 30(0.252) | 0.684 | 1.124(0.639-1.976) |
|  |  |  | AA | 95(0.725) | 89(0.748) |  | 1 (ref) |
|  |  | codominant (GA vs. AA) | GA | 118(0.554) | 131(0.595) | 0.383 | 0.844(0.576-1.236) |
|  |  |  | AA | 95(0.446) | 89(0.405) |  | 1 (ref) |
| *IL1B* | *rs2853550* | dominant (GG+GA vs. AA) | GG+GA | 246(0.984) | 248(0.992) | 0.686 | 0.496(0.090-2.733) |
|  |  |  | AA | 4(0.016) | 2(0.008) |  | 1 (ref) |
|  |  | recessive (GG vs. GA+AA) | GG | 207(0.828) | 205(0.820) | 0.814 | 1.057(0.667-1.675) |
|  |  |  | GA+AA | 43(0.172) | 45(0.180) |  | 1 (ref) |
|  |  | codominant (GG vs. AA) | GG | 207(0.981) | 205(0.990) | 0.685 | 0.505(0.091-2.787) |
|  |  |  | AA | 4(0.019) | 2(0.010) |  | 1 (ref) |
|  |  | codominant (GA vs. AA) | GA | 39(0.907) | 43(0.956) | 0.429 | 0.453(0.079-2.614) |
|  |  |  | AA | 4(0.093) | 2(0.044) |  | 1 (ref) |
| *IL1B* | *rs16944* | dominant (GG+GA vs. AA) | GG+GA | 197(0.788) | 200(0.800) | 0.740 | 0.929(0.602-1.434) |
|  |  |  | AA | 53(0.212) | 50(0.200) |  | 1 (ref) |
|  |  | recessive (GG vs. GA+AA) | GG | 74(0.296) | 61(0.244) | 0.190 | 1.303(0.876-1.936) |
|  |  |  | GA+AA | 176(0.704) | 189(0.745) |  | 1 (ref) |
|  |  | codominant (GG vs. AA) | GG | 74(0.583) | 61(0.550) | 0.607 | 1.144(0.684-1.913) |
|  |  |  | AA | 53(0.417) | 50(0.450) |  | 1 (ref) |
|  |  | codominant (GA vs. AA) | GA | 123(0.699) | 139(0.735) | 0.438 | 0.835(0.529-1.317) |
|  |  |  | AA | 53(0.301) | 50(0.265) |  | 1 (ref) |
| *IL1B* | *rs1143623* | dominant (CC+GC vs. GG) | CC+GC | 211(0.844) | 214(0.856) | 0.707 | 0.910(0.557-1.488) |
|  |  |  | GG | 39(0.156) | 36(0.144) |  | 1 (ref) |
|  |  | recessive (CC vs. GC+GG) | CC | 96(0.384) | 84(0.336) | 0.264 | 1.232(0.854-1.776) |
|  |  |  | GC+GG | 154(0.616) | 166(0.664) |  | 1 (ref) |
|  |  | codominant (CC vs. GG) | CC | 96(0.711) | 84(0.700) | 0.846 | 1.055(0.615-1.809) |
|  |  |  | GG | 39(0.289) | 36(0.300) |  | 1 (ref) |
|  |  | codominant (GC vs. GG) | GC | 115(0.747) | 130(0.783) | 0.443 | 0.817(0.487-1.371) |
|  |  |  | GG | 39(0.253) | 36(0.217) |  | 1 (ref) |
| *MAVS* | *rs7262903* | dominant (CC+AC vs. AA) | CC+AC | 246(0.984) | 247(0.988) | 1.000 | 0.747(0.165-3.372) |
|  |  |  | AA | 4(0.016) | 3(0.012) |  | 1 (ref) |
|  |  | recessive (CC vs. AC+AA) | CC | 199(0.796) | 185(0.740) | 0.138 | 1.371(0.903-2.082) |
|  |  |  | AC+AA | 51(0.204) | 65(0.260) |  | 1 (ref) |
|  |  | codominant (CC vs. AA) | CC | 199(0.980) | 185(0.984) | 1.000 | 0.807(0.178-3.653) |
|  |  |  | AA | 4(0.020) | 3(0.016) |  | 1 (ref) |
|  |  | codominant (AC vs. AA) | AC | 47(0.922) | 62(0.954) | 0.698 | 0.569(0.121-2.663) |
|  |  |  | AA | 4(0.078) | 3(0.046) |  | 1 (ref) |
| *MAVS* | *rs17857295* | dominant (CC+GC vs. GG) | CC+GC | 191(0.767) | 198(0.792) | 0.502 | 0.865(0.566-1.321) |
|  |  |  | GG | 58(0.233) | 52(0.208) |  | 1 (ref) |
|  |  | recessive (CC vs. GC+GG) | CC | 56(0.225) | 67(0.268) | 0.264 | 0.793(0.527-1.192) |
|  |  |  | GC+GG | 193(0.775) | 183(0.732) |  | 1 (ref) |
|  |  | codominant (CC vs. GG) | CC | 56(0.491) | 67(0.563) | 0.272 | 0.749(0.447-1.255) |
|  |  |  | GG | 58(0.509) | 52(0.437) |  | 1 (ref) |
|  |  | codominant (GC vs. GG) | GC | 135(0.699) | 131(0.716) | 0.727 | 0.924(0.592-1.442) |
|  |  |  | GG | 58(0.301) | 52(0.284) |  | 1 (ref) |
| *MAVS* | *rs6084497* | dominant (CC+TC vs. TT) | CC+TC | 218(0.872) | 211(0.844) | 0.370 | 1.259(0.760-2.085) |
|  |  |  | TT | 32(0.128) | 39(0.156) |  | 1 (ref) |
|  |  | recessive (CC vs. TC+TT) | CC | 101(0.404) | 88(0.352) | 0.231 | 1.248(0.869-1.793) |
|  |  |  | TC+TT | 149(0.596) | 162(0.648) |  | 1 (ref) |
|  |  | codominant (CC vs. TT) | CC | 101(0.759) | 88(0.693) | 0.229 | 1.399(0.809-2.419) |
|  |  |  | TT | 32(0.241) | 39(0.307) |  | 1 (ref) |
|  |  | codominant (TC vs. TT) | TC | 117(0.785) | 123(0.749) | 0.586 | 1.159(0.681-1.973) |
|  |  |  | TT | 32(0.215) | 39(0.241) |  | 1 (ref) |
| *MAVS* | *rs16989000* | dominant (AA+CA vs. CC) | AA+CA | 212(0.848) | 206(0.824) | 0.469 | 1.192(0.741-1.915) |
|  |  |  | CC | 38(0.1520 | 44(0.176) |  | 1 (ref) |
|  |  | recessive (AA vs. CA+CC) | AA | 91(0.364) | 74(0.296) | 0.106 | 1.361(0.936-1.979) |
|  |  |  | CA+CC | 159(0.636) | 176(0.704) |  | 1 (ref) |
|  |  | codominant (AA vs. CC) | AA | 91(0.705) | 74(0.627) | 0.192 | 1.424(0.837-2.423) |
|  |  |  | CC | 38(0.295) | 44(0.373) |  | 1 (ref) |
|  |  | codominant (CA vs. CC) | CA | 121(0.761) | 132(0.750) | 0.815 | 1.061(0.644-1.749) |
|  |  |  | CC | 38(0.239) | 44(0.250) |  | 1 (ref) |
| *MAVS* | *rs6515831* | dominant (CC+TC vs. TT) | CC+TC | 110(0.440) | 104(0.416) | 0.588 | 1.103(0.774-1.572) |
|  |  |  | TT | 140(0.560) | 146(0.584) |  | 1 (ref) |
|  |  | recessive (CC vs. TC+TT) | CC | 12(0.048) | 9(0.036) | 0.504 | 1.350(0.559-3.264) |
|  |  |  | TC+TT | 238(0.952) | 241(0.964) |  | 1 (ref) |
|  |  | codominant (CC vs. TT) | CC | 12(0.079) | 9(0.058) | 0.469 | 1.390(0.568-3.402) |
|  |  |  | TT | 140(0.921) | 146(0.942) |  | 1 (ref) |
|  |  | codominant (TC vs. TT) | TC | 98(0.412) | 95(0.394) | 0.695 | 1.076(0.747-1.550) |
|  |  |  | TT | 140(0.588) | 146(0.606) |  | 1 (ref) |
| *MAVS* | *rs57173648* | dominant (TT+TC vs. CC) | TT+TC | 30(0.120) | 32(0.128) | 0.786 | 0.929(0.546-1.582) |
|  |  |  | CC | 220(0.880) | 218(0.872) |  | 1 (ref) |
|  |  | recessive (TT vs. TC+CC) | TT | 2(0.008) | 0(0.000) | 0.499 | 0.992(0.981-1.003) |
|  |  |  | TC+CC | 248(0.992) | 250(1.000) |  | 1 (ref) |
|  |  | codominant (TT vs. CC) | TT | 2(0.009) | 0(0.000) | 0.499 | 0.991(0.979-1.003) |
|  |  |  | CC | 220(0.991) | 218(1.000) |  | 1 (ref) |
|  |  | codominant (TC vs. CC) | TC | 28(0.113) | 32(0.128) | 0.605 | 0.867(0.505-1.489) |
|  |  |  | CC | 220(0.887) | 218(0.872) |  | 1 (ref) |
| *MAVS* | *rs867335* | dominant (TT+AT vs. AA) | TT+AT | 231(0.928) | 240(0.960) | 0.117 | 0.535(0.242-1.183) |
|  |  |  | AA | 18(0.072) | 10(0.040) |  | 1 (ref) |
|  |  | recessive (TT vs. AT+AA) | TT | 141(0.566) | 125(0.500) | 0.138 | 1.306(0.918-1.857) |
|  |  |  | AT+AA | 108(0.434) | 125(0.500) |  | 1 (ref) |
|  |  | codominant (TT vs. AA) | TT | 141(0.887) | 125(0.926) | 0.255 | 0.627(0.279-1.408) |
|  |  |  | AA | 18(0.113) | 10(0.074) |  | 1 (ref) |
|  |  | codominant (AT vs. AA) | AT | 90(0.833) | 115(0.920) | **0.042** | 2.300(1.012-5.226) |
|  |  |  | **AA** | 18(0.167) | 10(0.080) |  |  |
| *JAK1* | *rs7531799* | dominant (TT+TC vs. CC) | TT+TC | 178(0.712) | 180(0.720) | 0.843 | 0.961(0.652-1.418) |
|  |  |  | CC | 72(0.288) | 70(0.280) |  | 1 (ref) |
|  |  | recessive (TT vs. TC+CC) | TT | 59(0.236) | 56(0.224) | 0.750 | 1.070(0.705-1.623) |
|  |  |  | TC+CC | 191(0.764) | 194(0.776) |  | 1 (ref) |
|  |  | codominant (TT vs. CC) | TT | 59(0.450) | 56(0.444) | 0.924 | 1.024(0.626-1.675) |
|  |  |  | CC | 72(0.550) | 70(0.556) |  | 1 (ref) |
|  |  | codominant (TC vs. CC) | TC | 119(0.623) | 124(0.639) | 0.743 | 0.933(0.617-1.412) |
|  |  |  | CC | 72(0.377) | 70(0.361) |  | 1 (ref) |
| *JAK1* | *rs4244165* | dominant (TT+TG vs. GG) | TT+TG | 144(0.578) | 143(0.572) | 0.887 | 1.026(0.720-1.464) |
|  |  |  | GG | 105(0.422) | 107(0.428) |  | 1 (ref) |
|  |  | recessive (TT vs. TG+GG) | TT | 27(0.108) | 25(0.010) | 0.758 | 1.095(0.616-1.945) |
|  |  |  | TG+GG | 222(0.892) | 225(0.090) |  | 1 (ref) |
|  |  | codominant (TT vs. GG) | TT | 27(0.205) | 25(0.189) | 0.757 | 1.101(0.600-2.019) |
|  |  |  | GG | 105(0.795) | 107(0.811) |  | 1 (ref) |
|  |  | codominant (TG vs. GG) | TG | 117(0.527) | 118(0.524) | 0.956 | 1.010(0.697-1.465) |
|  |  |  | GG | 105(0.473) | 107(0.476) |  | 1 (ref) |
| *JAK1* | *rs1039125* | dominant (CC+TC vs. TT) | CC+TC | 166(0.664) | 161(0.644) | 0.638 | 1.092(0.756-1.579) |
|  |  |  | TT | 84(0.336) | 89(0.356) |  | 1 (ref) |
|  |  | recessive (CC vs. TC+TT) | CC | 45(0.180) | 41(0.164) | 0.635 | 1.119(0.703-1.781) |
|  |  |  | TC+TT | 205(0.820) | 209(0.836) |  | 1 (ref) |
|  |  | codominant (CC vs. TT) | CC | 45(0.349) | 41(0.315) | 0.568 | 1.163(0.693-1.951) |
|  |  |  | TT | 84(0.651) | 89(0.685) |  | 1 (ref) |
|  |  | codominant (TC vs. TT) | TC | 121(0.590) | 120(0.574) | 0.740 | 1.068(0.723-1.579) |
|  |  |  | TT | 84(0.410) | 89(0.426) |  | 1 (ref) |
| *JAK1* | *rs56818621* | dominant (GG+GC vs. CC) | GG+GC | 154(0.618) | 147(0.588) | 0.487 | 1.136(0.793-1.626) |
|  |  |  | CC | 95(0.382) | 103(0.412) |  | 1 (ref) |
|  |  | recessive (GG vs. GC+CC) | GG | 40(0.161) | 36(0.144) | 0.605 | 1.138(0.698-1.855) |
|  |  |  | GC+CC | 209(0.839) | 214(0.856) |  | 1 (ref) |
|  |  | codominant (GG vs. CC) | GG | 40(0.296) | 36(0.259) | 0.490 | 1.205(0.709-2.046) |
|  |  |  | CC | 95(0.704) | 103(0.741) |  | 1 (ref) |
|  |  | codominant (GC vs. CC) | GC | 114(0.545) | 111(0.519) | 0.581 | 1.114(0.760-1.632) |
|  |  |  | CC | 95(0.455) | 103(0.481) |  | 1 (ref) |
| *JAK1* | *rs11579758* | dominant (AA+GA vs. GG) | AA+GA | 146(0.584) | 151(0.604) | 0.649 | 0.920(0.644-1.315) |
|  |  |  | GG | 104(0.416) | 99(0.396) |  | 1 (ref) |
|  |  | recessive (AA vs. GA+GG) | AA | 31(0.207) | 30(0.020) | 0.886 | 1.042(0.594-1.829) |
|  |  |  | GA+GG | 119(0.793) | 120(0.080) |  | 1 (ref) |
|  |  | codominant (AA vs. GG) | AA | 31(0.230) | 30(0.233) | 0.955 | 0.984(0.555-1.744) |
|  |  |  | GG | 104(0.770) | 99(0.767) |  | 1 (ref) |
|  |  | codominant (GA vs. GG) | GA | 115(0.525) | 121(0.550) | 0.601 | 0.905(0.622-1.317) |
|  |  |  | GG | 104(0.475) | 99(0.450) |  | 1 (ref) |
| *JAK1* | *rs567354* | dominant (GG+GA vs. AA) | GG+GA | 217(0.868) | 210(0.840) | 0.375 | 1.253(0.761-2.062) |
|  |  |  | AA | 33(0.132) | 40(0.160) |  | 1 (ref) |
|  |  | recessive (GG vs. GA+AA) | GG | 84(0.336) | 83(0.332) | 0.924 | 1.018(0.702-1.477) |
|  |  |  | GA+AA | 166(0.664) | 167(0.668) |  | 1 (ref) |
|  |  | codominant (GG vs. AA) | GG | 84(0.718) | 83(0.675) | 0.468 | 1.227(0.707-2.130) |
|  |  |  | AA | 33(0.282) | 40(0.325) |  | 1 (ref) |
|  |  | codominant (GA vs. AA) | GA | 133(0.801) | 127(0.760) | 0.369 | 1.269(0.754-2.138) |
|  |  |  | AA | 33(0.199) | 40(0.240) |  | 1 (ref) |
| *JAK1* | *rs490178* | dominant (GG+GA vs. AA) | GG+GA | 123(0.494) | 119(0.476) | 0.688 | 1.075(0.756-1.527) |
|  |  |  | AA | 126(0.506) | 131(0.524) |  | 1 (ref) |
|  |  | recessive (GG vs. GA+AA) | GG | 16(0.064) | 16(0.064) | 0.991 | 1.004(0.491-2.056) |
|  |  |  | GA+AA | 233(0.936) | 234(0.936) |  | 1 (ref) |
|  |  | codominant (GG vs. AA) | GG | 16(0.113) | 16(0.109) | 0.917 | 1.040(0.499-2.168) |
|  |  |  | AA | 126(0.887) | 131(0.891) |  | 1 (ref) |
|  |  | codominant (GA vs. AA) | GA | 107(0.459) | 103(0.440) | 0.679 | 1.080(0.750-1.555) |
|  |  |  | AA | 126(0.541) | 131(0.560) |  | 1 (ref) |
| *JAK1* | *rs705509* | dominant (AA+GA vs. GG) | AA+GA | 170(0.683) | 167(0.668) | 0.725 | 1.070(0.735-1.556) |
|  |  |  | GG | 79(0.317) | 83(0.332) |  | 1 (ref) |
|  |  | recessive (AA vs. GA+GG) | AA | 50(0.201) | 43(0.172) | 0.409 | 1.210(0.770-1.900) |
|  |  |  | GA+GG | 199(0.799) | 207(0.828) |  | 1 (ref) |
|  |  | codominant (AA vs. GG) | AA | 50(0.388) | 43(0.341) | 0.442 | 1.222(0.733-2.036) |
|  |  |  | GG | 79(0.612) | 83(0.659) |  | 1 (ref) |
|  |  | codominant (GA vs. GG) | GA | 120(0.603) | 124(0.599) | 0.935 | 1.017(0.683-1.513) |
|  |  |  | GG | 79(0.397) | 83(0.401) |  | 1 (ref) |
| *JAK1* | *rs489500* | dominant (CC+GC vs. GG) | CC+GC | 227(0.912) | 219(0.876) | 0.196 | 1.461(0.820-2.601) |
|  |  |  | GG | 22(0.088) | 31(0.124) |  | 1 (ref) |
|  |  | recessive (CC vs. GC+GG) | CC | 114(0.458) | 114(0.456) | 0.967 | 1.007(0.708-1.433) |
|  |  |  | GC+GG | 135(0.542) | 136(0.544) |  | 1 (ref) |
|  |  | codominant (CC vs. GG) | CC | 114(0.838) | 114(0.786) | 0.265 | 1.409(0.770-2.580) |
|  |  |  | GG | 22(0.162) | 31(0.214) |  | 1 (ref) |
|  |  | codominant (GC vs. GG) | GC | 113(0.837) | 105(0.772) | 0.178 | 1.516(0.826-2.784) |
|  |  |  | GG | 22(0.163) | 31(0.228) |  | 1 (ref) |
| *JAK1* | *rs310241* | dominant (AA+GA vs. GG) | AA+GA | 234(0.940) | 228(0.912) | 0.237 | 1.505(0.762-2.975) |
|  |  |  | GG | 15(0.060) | 22(0.088) |  | 1 (ref) |
|  |  | recessive (AA vs. GA+GG) | AA | 140(0.562) | 134(0.536) | 0.556 | 1.112(0.781-1.582) |
|  |  |  | GA+GG | 109(0.438) | 116(0.464) |  | 1 (ref) |
|  |  | codominant (AA vs. GG) | AA | 140(0.907) | 134(0.859) | 0.228 | 1.532(0.763-3.079) |
|  |  |  | GG | 15(0.093) | 22(0.141) |  | 1 (ref) |
|  |  | codominant (GA vs. GG) | GA | 94(0.862) | 94(0.810) | 0.293 | 1.467(0.717-3.001) |
|  |  |  | GG | 15(0.138) | 22(0.190) |  | 1 (ref) |
| ^a^Results are shown as n (frequency). | | | | | | | |
| Bold type indicates statistical significance (*p* < 0.05). | | | | | | | |
