# Supplementary material for: Genetic Variants in the NOD-like Receptor Signaling Pathway Are Associated with HIV-1/AIDS in a Northern Chinese Population
Source: Int J Mol Sci. 2025 Apr 8;26(8):3484. doi: 10.3390/ijms26083484 (PMC12026778; doi:10.3390/ijms26083484)
Supplement: Supplementary file 1 [file ijms-26-03484-s001.zip › Supplementary_Table_S9_R3.docx]

| **Table S9. Association between the number of risk alleles and AIDS stage** | | | | | | |
| --- | --- | --- | --- | --- | --- | --- |
| Risk allele(n) | Case with risk allele | Case without risk allele | Control with risk allele | Control without risk allele | *p* value | OR (95%CI) |
| 1 | 35 | 9 | 34 | 7 | 0.690 | 0.801(0.268-2.393) |
| 2 | 71 | 9 | 67 | 7 | 0.716 | 0.824(0.291-2.338) |
| 3 | 71 | 9 | 94 | 7 | 0.309 | 0.587(0.209-1.653) |
| 4 | 58 | 9 | 42 | 7 | 0.895 | 1.074(0.370-3.114) |
| 5 | 4 | 9 | 5 | 7 | 0.571 | 0.622(0.120-3.222) |
| 6 | 1 | 9 | 0 | 7 | 1.000 | 0.900(0.732-1.107) |
