# Supplementary material for: Genetic Variants in the NOD-like Receptor Signaling Pathway Are Associated with HIV-1/AIDS in a Northern Chinese Population
Source: Int J Mol Sci. 2025 Apr 8;26(8):3484. doi: 10.3390/ijms26083484 (PMC12026778; doi:10.3390/ijms26083484)
Supplement: Supplementary file 1 [file ijms-26-03484-s001.zip › Supplementary_ Table_S1.docx]

| **Table S1. The detailed information of 37 candidate SNPs** | | | | | | | | | | |
| --- | --- | --- | --- | --- | --- | --- | --- | --- | --- | --- |
| ID | Gene | SNPinfo | eQTL | GWAS4D | | | CADD | PolyP | RegulomeDB | |
|  |  |  |  | FitCons2 | FUNLDA | GenoNet |  |  | rank | possibility |
| *rs530537* | *CASP1* | - | N | 0.144 | 0.065 | 0.860 | 1.191 | 0.613 | 1f | 0.554 |
| *rs10774671* | *OAS1* | - | Y | 0.917 | 0.000 | 0.164 | 4.237 | 1.927 | 1f | 0.554 |
| *rs1131454* | *OAS1* | Splicing;nsSNP | Y | 0.751 | 0.174 | 0.433 | 2.411 | 1.602 | 1f | 0.554 |
| *rs2066804* | *STAT1* | - | Y | 0.328 | 0.000 | 0.087 | 0.706 | 0.077 | 1f | 0.554 |
| *rs1467199* | *STAT1* | TFBS | N | 0.124 | 0.000 | 0.709 | 8.368 | 0.510 | 1f | 0.553 |
| *rs11551202* | *GSDMD* | - | Y | 0.751 | 0.003 | 0.232 | 5.110 | 0.108 | 1f | 0.554 |
| *rs1545536* | *GSDMD* | - | Y | 0.281 | 0.000 | 0.148 | 14.640 | 1.282 | 1f | 0.783 |
| *rs7834318* | *GSDMD* | TBFS | Y | 0.146 | 0.001 | 0.301 | 2.179 | 0.811 | 1f | 0.554 |
| *rs549908* | *IL18* | Splicing | Y | 0.751 | 0.000 | 0.059 | 6.128 | 0.418 | 1f | 0.223 |
| *rs360719* | *IL18* | TFBS | - | - | - | - | - | - | 1f | 0.667 |
| *rs1946518* | *IL18* | TFBS | Y | 0.146 | 0.798 | 0.470 | 0.270 | 0.087 | 1f | 0.677 |
| *rs10754558* | *NLRP3* | miRNA | N | 0.140 | 0.086 | 0.854 | 6.012 | 0.307 | 1f | 0.554 |
| *rs4612666* | *NLRP3* | - | N | 0.144 | 0.998 | 0.660 | -0.001 | 1.605 | 1f | 0.554 |
| *rs3806265* | *NLRP3* | - | N | 0.000 | 0.243 | 0.010 | 0.247 | 1.298 | 1f | 0.554 |
| *rs1539019* | *NLRP3* | - | N | 0.144 | 0.852 | 0.701 | 1.323 | 0.199 | 7 | 0.184 |
| *rs4848306* | *IL1B* | TFBS | N | 0.146 | 0.998 | 0.238 | 0.430 | 0.134 | 1f | 0.667 |
| *rs3136558* | *IL1B* | - | N | 0.996 | 1.000 | 0.158 | 2.555 | 0.777 | 1f | 0.667 |
| *rs2853550* | *IL1B* | - | N | 0.004 | 0.560 | 0.982 | 9.204 | 0.199 | 1f | 0.554 |
| *rs16944* | *IL1B* | TFBS | - | - | - | - | - | - | 1f | 0.195 |
| *rs1143623* | *IL1B* | TFBS | N | 0.146 | 0.047 | 0.791 | 2.595 | 0.200 | 1f | 0.223 |
| *rs7262903* | *MAVS* | Splicing;nsSNP | N | 0.465 | 0.001 | 0.162 | 23.000 | 0.263 | 1f | 0.553 |
| *rs17857295* | *MAVS* | Splicing;nsSNP | N | 0.707 | 0.000 | 0.176 | 8.331 | 1.093 | 1f | 0.554 |
| *rs6084497* | *MAVS* | - | Y | 0.133 | 0.000 | 0.209 | 12.320 | 0.027 | 1f | 0.667 |
| *rs16989000* | *MAVS* | miRNA | Y | 0.270 | 0.003 | 0.141 | 1.717 | 0.959 | 1f | 0.554 |
| *rs6515831* | *MAVS* | miRNA | Y | 0.270 | 0.000 | 0.198 | 5.449 | 0.994 | 1b | 0.140 |
| *rs57173648* | *MAVS* | miRNA | N | 0.408 | 0.000 | 0.082 | 13.040 | 2.220 | 4 | 0.609 |
| *rs867335* | *MAVS* | miRNA | N | 0.124 | 0.050 | 0.293 | 13.840 | 1.796 | 1b | 0.980 |
| *rs7531799* | *JAK1* | - | Y | 0.133 | 0.007 | 0.499 | 14.660 | 0.305 | 1d | 0.325 |
| *rs4244165* | *JAK1* | - | Y | 0.144 | 0.007 | 0.952 | 10.490 | 0.524 | 1f | 0.554 |
| *rs1039125* | *JAK1* | - | Y | 0.140 | 0.064 | 1.000 | 16.290 | 1.259 | 1b | 0.681 |
| *rs56818621* | *JAK1* | TFBS | Y | 0.124 | 0.003 | 0.156 | 1.808 | 0.127 | 1f | 0.554 |
| *rs11579758* | *JAK1* | - | N | 0.065 | 0.000 | 0.044 | 1.762 | 0.110 | 6 | 0.220 |
| *rs567354* | *JAK1* | - | Y | 0.065 | 0.000 | 0.056 | 9.773 | 1.981 | 1f | 0.667 |
| *rs490178* | *JAK1* | - | N | 0.140 | 1.000 | 1.000 | 5.977 | 0.201 | 1f | 0.667 |
| *rs705509* | *JAK1* | - | N | 0.065 | 0.576 | 0.393 | 8.997 | 0.299 | 1f | 0.554 |
| *rs489500* | *JAK1* | TFBS | N | 0.146 | 0.002 | 0.557 | 6.566 | 0.118 | 1b | 0.772 |
| *rs310241* | *JAK1* | - | N | 0.144 | 0.000 | 0.088 | 7.176 | 0.156 | 1b | 0.640 |
