# Supplementary material for: Genetic Variants in the NOD-like Receptor Signaling Pathway Are Associated with HIV-1/AIDS in a Northern Chinese Population
Source: Int J Mol Sci. 2025 Apr 8;26(8):3484. doi: 10.3390/ijms26083484 (PMC12026778; doi:10.3390/ijms26083484)
Supplement: Supplementary file 1 [file ijms-26-03484-s001.zip › Supplementary_Table_S2_R3.docx]

| **Table S2. Genotype distributions of the 37 candidate SNPs and the HWE test** | | | | | | | | | | |
| --- | --- | --- | --- | --- | --- | --- | --- | --- | --- | --- |
| SNP | Gene | Risk allele^*^ | Case genotype | | | Control genotype | | | *x*^2^ | HWE *p* value |
|  |  |  | 11^a^ | 12^a^ | 22^a^ | 11^a^ | 12^a^ | 22^a^ |  |  |
| *rs530537* | *CASP1* | T | 305 | 173 | 22 | 300 | 177 | 23 | 0.23 | 0.63 |
| *rs2066804* | *STAT1* | G | 151 | 259 | 90 | 144 | 247 | 109 | 0.03 | 0.87 |
| *rs1467199* | *STAT1* | C | 123 | 256 | 121 | 142 | 238 | 120 | 1.07 | 0.30 |
| *rs10774671* | *OAS1* | G | 50 | 195 | 255 | 34 | 212 | 254 | 1.33 | 0.25 |
| *rs1131454* | *OAS1* | A | 122 | 232 | 146 | 126 | 227 | 147 | 4.09 | 0.05 |
| *rs549908* | *IL18* | T | 393 | 104 | 3 | 373 | 122 | 5 | 2.10 | 0.15 |
| *rs360719* | *IL18* | A | 2 | 107 | 390 | 5 | 122 | 373 | 2.10 | 0.15 |
| *rs1946518* | *IL18* | G | 106 | 258 | 136 | 129 | 247 | 124 | 0.07 | 0.79 |
| *rs11551202* | *GSDMD* | A | 380 | 109 | 10 | 386 | 108 | 6 | 0.26 | 0.61 |
| *rs1545536* | *GSDMD* | C | 91 | 255 | 153 | 102 | 249 | 149 | 0.01 | 0.91 |
| *rs7834318* | *GSDMD* | A | 165 | 245 | 90 | 148 | 267 | 85 | 3.63 | 0.06 |
| *rs10754558* | *NLRP3* | C | 112 | 229 | 159 | 107 | 251 | 142 | 0.04 | 0.84 |
| *rs4612666* | *NLRP3* | T | 87 | 273 | 140 | 91 | 239 | 170 | 0.19 | 0.66 |
| *rs3806265* | *NLRP3* | C | 135 | 265 | 100 | 150 | 241 | 109 | 0.43 | 0.51 |
| *rs1539019* | *NLRP3* | A | 106 | 246 | 148 | 92 | 254 | 154 | 0.51 | 0.48 |
| *rs4848306* | *IL1B* | A | 123 | 256 | 120 | 137 | 242 | 121 | 0.48 | 0.49 |
| *rs3136558* | *IL1B* | A | 66 | 249 | 184 | 79 | 248 | 173 | 0.40 | 0.53 |
| *rs2853550* | *IL1B* | G | 412 | 82 | 6 | 399 | 99 | 2 | 2.56 | 0.11 |
| *rs16944* | *IL1B* | G | 135 | 262 | 103 | 135 | 241 | 124 | 0.63 | 0.43 |
| *rs1143623* | *IL1B* | C | 75 | 245 | 180 | 91 | 240 | 169 | 0.13 | 0.72 |
| *rs7262903* | *MAVS* | A | 7 | 109 | 384 | 6 | 92 | 402 | 0.08 | 0.78 |
| *rs17857295* | *MAVS* | C | 110 | 266 | 123 | 137 | 238 | 125 | 1.13 | 0.29 |
| *rs6084497* | *MAVS* | T | 71 | 240 | 189 | 61 | 219 | 220 | 0.32 | 0.57 |
| *rs16989000* | *MAVS* | C | 165 | 253 | 82 | 196 | 234 | 70 | 0.00 | 0.99 |
| *rs6515831* | *MAVS* | T | 286 | 193 | 21 | 289 | 179 | 32 | 0.36 | 0.55 |
| *rs57173648* | *MAVS* | T | 2 | 60 | 438 | 1 | 62 | 437 | 0.61 | 0.43 |
| *rs867335* | *MAVS* | A | 266 | 205 | 28 | 288 | 183 | 29 | 0.00 | 0.99 |
| *rs7531799* | *JAK1* | T | 115 | 243 | 142 | 106 | 249 | 145 | 0.00 | 0.96 |
| *rs4244165* | *JAK1* | T | 52 | 235 | 212 | 59 | 197 | 244 | 3.79 | 0.05 |
| *rs1039125* | *JAK1* | T | 173 | 241 | 86 | 162 | 245 | 93 | 0.00 | 0.98 |
| *rs56818621* | *JAK1* | C | 76 | 225 | 198 | 82 | 238 | 180 | 0.05 | 0.82 |
| *rs11579758* | *JAK1* | A | 203 | 236 | 61 | 221 | 222 | 57 | 0.01 | 0.91 |
| *rs567354* | *JAK1* | A | 167 | 260 | 73 | 192 | 228 | 80 | 0.79 | 0.37 |
| *rs490178* | *JAK1* | G | 32 | 210 | 257 | 35 | 187 | 278 | 0.21 | 0.64 |
| *rs705509* | *JAK1* | G | 162 | 244 | 93 | 144 | 264 | 92 | 2.28 | 0.13 |
| *rs489500* | *JAK1* | C | 53 | 218 | 228 | 56 | 217 | 227 | 0.14 | 0.70 |
| *rs310241* | *JAK1* | A | 37 | 188 | 274 | 44 | 207 | 249 | 0.01 | 0.92 |
| Risk alleles refer to alleles that have a higher frequency in the case group compared to the control group; HWE Hardy-Weinberg equilibrium, SNP single nucleotide polymorphism. | | | | | | | | | | |
| ^a^1: risk allele, 2: non-risk allele. | | | | | | | | | | |
