# Supplementary material for: Genetic Variants in the NOD-like Receptor Signaling Pathway Are Associated with HIV-1/AIDS in a Northern Chinese Population
Source: Int J Mol Sci. 2025 Apr 8;26(8):3484. doi: 10.3390/ijms26083484 (PMC12026778; doi:10.3390/ijms26083484)
Supplement: Supplementary file 1 [file ijms-26-03484-s001.zip › Supplementary_Table_S3_R3.docx]

| **Table S3. Association between alleles of 37 candidate SNPs and HIV-1 infection** | | | | | | | |
| --- | --- | --- | --- | --- | --- | --- | --- |
| Gene | SNP | Major/Minor allele | Risk allele | Risk allele frequency | | *p* value | OR (95%CI) |
|  |  |  |  | Case^a^ | Control^a^ |  |  |
| *CASP1* | *rs530537* | T/C | T | 783(0.783) | 777(0.777) | 0.746 | 1.036(0.838-1.280) |
| *STAT1* | *rs2066804* | G/A | G | 561(0.561) | 535(0.535) | 0.243 | 1.111(0.931-1.325) |
| *STAT1* | *rs1467199* | G/C | C | 498(0.498) | 478(0.478) | 0.371 | 1.083(0.909-1.291) |
| *OAS1* | *rs10774671* | A/G | G | 295(0.295) | 280(0.280) | 0.459 | 1.076(0.886-1.306) |
| *OAS1* | *rs1131454* | A/G | A | 524(0.524) | 521(0.522) | 0.930 | 1.008(0.846-1.201) |
| *IL18* | *rs549908* | T/G | T | 890(0.890) | 868(0.868) | 0.131 | 1.230(0.940-1.611) |
| *IL18* | *rs360719* | A/G | A | 887(0.889) | 868(0.868) | 0.155 | 1.215(0.928-1.591) |
| *IL18* | *rs1946518* | G/T | G | 530(0.530) | 495(0.495) | 0.117 | 1.150(0.965-1.371) |
| *GSDMD* | *rs11551202* | G/A | A | 129(0.129) | 120(0.120) | 0.531 | 1.089(0.835-1.420) |
| *GSDMD* | *rs1545536* | C/T | C | 561(0.562) | 547(0.547) | 0.496 | 1.063(0.891-1.268) |
| *GSDMD* | *rs7834318* | A/C | A | 575(0.575) | 563(0.563) | 0.588 | 1.050(0.880-1.254) |
| *NLRP3* | *rs10754558* | C/G | C | 547(0.547) | 535(0.535) | 0.590 | 1.050(0.880-1.251) |
| *NLRP3* | *rs4612666* | C/T | T | 447(0.447) | 421(0.421) | 0.241 | 1.112(0.931-1.327) |
| *NLRP3* | *rs3806265* | T/C | C | 465(0.465) | 459(0.459) | 0.788 | 1.024(0.859-1.221) |
| *NLRP3* | *rs1539019* | C/A | A | 458(0.458) | 438(0.438) | 0.368 | 1.084(0.909-1.293) |
| *IL1B* | *rs4848306* | G/A | A | 496(0.497) | 484(0.484) | 0.561 | 1.053(0.884-1.255) |
| *IL1B* | *rs3136558* | A/G | A | 617(0.618) | 594(0.594) | 0.258 | 1.107(0.925-1.325) |
| *IL1B* | *rs2853550* | G/A | G | 906(0.906) | 897(0.897) | 0.499 | 1.107(0.824-1.486) |
| *IL1B* | *rs16944* | G/A | G | 532(0.532) | 511(0.511) | 0.347 | 1.088(0.913-1.297) |
| *IL1B* | *rs1143623* | C/G | C | 605(0.605) | 578(0.578) | 0.219 | 1.118(0.936-1.337) |
| *MAVS* | *rs7262903* | C/A | A | 123(0.123) | 104(0.104) | 0.180 | 1.208(0.916-1.594) |
| *MAVS* | *rs17857295* | C/G | C | 512(0.513) | 488(0.488) | 0.263 | 1.105(0.927-1.317) |
| *MAVS* | *rs6084497* | C/T | T | 382(0.382) | 341(0.341) | 0.056 | 1.195(0.995-1.434) |
| *MAVS* | *rs16989000* | A/C | C | 417(0.417) | 374(0.374) | **0.049** | 1.197(1.001-1.433) |
| *MAVS* | *rs6515831* | T/C | T | 765(0.765) | 757(0.757) | 0.675 | 1.045(0.851-1.283) |
| *MAVS* | *rs57173648* | C/T | T | 64(0.064) | 64(0.064) | 1.000 | 1.000(0.699-1.431) |
| *MAVS* | *rs867335* | T/A | A | 261(0.262) | 241(0.241) | 0.290 | 1.115(0.911-1.365) |
| *JAK1* | *rs7531799* | C/T | T | 473(0.473) | 461(0.461) | 0.591 | 1.049(0.880-1.251) |
| *JAK1* | *rs4244165* | G/T | T | 339(0.340) | 315(0.315) | 0.240 | 1.119(0.928-1.349) |
| *JAK1* | *rs1039125* | T/C | T | 587(0.587) | 569(0.569) | 0.415 | 1.077(0.901-1.286) |
| *JAK1* | *rs56818621* | C/G | C | 621(0.622) | 598(0.598) | 0.267 | 1.107(0.925-1.326) |
| *JAK1* | *rs11579758* | G/A | A | 358(0.358) | 336(0.336) | 0.301 | 1.102(0.917-1.325) |
| *JAK1* | *rs567354* | G/A | A | 406(0.406) | 388(0.388) | 0.411 | 1.078(0.901-1.290) |
| *JAK1* | *rs490178* | A/G | G | 274(0.275) | 257(0.257) | 0.375 | 1.094(0.897-1.335) |
| *JAK1* | *rs705509* | G/A | G | 568(0.569) | 552(0.552) | 0.440 | 1.072(0.898-1.279) |
| *JAK1* | *rs489500* | C/G | C | 674(0.675) | 671(0.671) | 0.836 | 1.020(0.846-1.230) |
| *JAK1* | *rs310241* | A/G | A | 736(0.738) | 705(0.705) | 0.106 | 1.175(0.966-1.430) |
| ^a^Results are shown as *n* (frequency). | | | | | | | |
| Bold type indicates statistical significance (*p* < 0.05). | | | | | | | |
