# Supplementary material for: Genetic Variants in the NOD-like Receptor Signaling Pathway Are Associated with HIV-1/AIDS in a Northern Chinese Population
Source: Int J Mol Sci. 2025 Apr 8;26(8):3484. doi: 10.3390/ijms26083484 (PMC12026778; doi:10.3390/ijms26083484)
Supplement: Supplementary file 1 [file ijms-26-03484-s001.zip › Supplementary_Table_S4.docx]

**Table S4. Association between genotypes of 37 candidate SNPs and HIV-1 infection**

| Gene | SNP | Genetic models | Genotype | Case^a^ | Control^a^ | *p* value | OR (95%CI) |
| --- | --- | --- | --- | --- | --- | --- | --- |
| *CASP1* | *rs530537* | dominant (TT+TC vs. CC) | TT+TC | 478(0.956) | 477(0.954) | 0.897 | 1.048(0.576-1.905) |
|  |  |  | CC | 22(0.044) | 23(0.046) |  | 1 (ref) |
|  |  | recessive (TT vs. TC+CC) | TT | 305(0.610) | 300(0.600) | 0.746 | 1.043(0.809-1.344) |
|  |  |  | TC+CC | 195(0.390) | 200(0.400) |  | 1 (ref) |
|  |  | codominant (TT vs. CC) | TT | 305(0.933) | 300(0.929) | 0.844 | 1.063(0.580-1.948) |
|  |  |  | CC | 22(0.067) | 23(0.071) |  | 1 (ref) |
|  |  | codominant (TC vs. CC) | TC | 173(0.887) | 177(0.885) | 0.946 | 1.022(0.549-1.901) |
|  |  |  | CC | 22(0.113) | 23(0.115) |  | 1 (ref) |
| *STAT1* | *rs2066804* | dominant (GG+GA vs. AA) | GG+GA | 410(0.820) | 391(0.782) | 0.132 | 1.270(0.930-1.734) |
|  |  |  | AA | 90(0.180) | 109(0.218) |  | 1 (ref) |
|  |  | recessive (GG vs. GA+AA) | GG | 151(0.302) | 144(0.288) | 0.627 | 1.070(0.815-1.404) |
|  |  |  | GA+AA | 349(0.698) | 356(0.712) |  | 1 (ref) |
|  |  | codominant (GG vs. AA) | GG | 151(0.627) | 144(0.569) | 0.194 | 1.270(0.885-1.821) |
|  |  |  | AA | 90(0.373) | 109(0.431) |  | 1 (ref) |
|  |  | codominant (GA vs. AA) | GA | 259(0.742) | 247(0.694) | 0.154 | 1.270(0.914-1.765) |
|  |  |  | AA | 90(0.258) | 109(0.306) |  | 1 (ref) |
| *STAT1* | *rs1467199* | dominant (CC+GC vs. GG) | CC+GC | 377(0.754) | 358(0.716) | 0.173 | 1.216(0.918-1.611) |
|  |  |  | GG | 123(0.246) | 142(0.284) |  | 1 (ref) |
|  |  | recessive (CC vs. GC+GG) | CC | 121(0.242) | 120(0.240) | 0.941 | 1.011(0.757-1.351) |
|  |  |  | GC+GG | 379(0.758) | 380(0.760) |  | 1 (ref) |
|  |  | codominant (CC vs. GG) | CC | 121(0.496) | 120(0.458) | 0.394 | 1.164(0.821-1.651) |
|  |  |  | GG | 123(0.504) | 142(0.542) |  | 1 (ref) |
|  |  | codominant (GC vs. GG) | GC | 256(0.675) | 238(0.626) | 0.156 | 1.242(0.921-1.675) |
|  |  |  | GG | 123(0.325) | 142(0.374) |  | 1 (ref) |
| *OAS1* | *rs10774671* | dominant (GG+GA vs. AA) | GG+GA | 245(0.490) | 246(0.492) | 0.950 | 0.992(0.774-1.271) |
|  |  |  | AA | 255(0.510) | 254(0.508) |  | 1 (ref) |
|  |  | recessive (GG vs. GA+AA) | GG | 50(0.100) | 34(0.068) | 0.068 | 1.523(0.967-2.399) |
|  |  |  | GA+AA | 450(0.900) | 466(0.932) |  | 1 (ref) |
|  |  | codominant (GG vs. AA) | GG | 50(0.164) | 34(0.118) | 0.109 | 1.465(0.916-2.341) |
|  |  |  | AA | 255(0.836) | 254(0.882) |  | 1 (ref) |
|  |  | codominant (GA vs. AA) | GA | 195(0.433) | 212(0.455) | 0.511 | 0.916(0.706-1.189) |
|  |  |  | AA | 255(0.567) | 254(0.545) |  | 1 (ref) |
| *OAS1* | *rs1131454* | dominant (AA+AG vs. GG) | AA+AG | 378(0.756) | 374(0.749) | 0.812 | 1.036(0.777-1.380) |
|  |  |  | GG | 122(0.244) | 125(0.251) |  | 1 (ref) |
|  |  | recessive (AA vs. AG+GG) | AA | 146(0.292) | 147(0.295) | 0.928 | 0.988(0.752-1.297) |
|  |  |  | AG+GG | 354(0.708) | 352(0.705) |  | 1 (ref) |
|  |  | codominant (AA vs. GG) | AA | 146(0.545) | 147(0.540) | 0.919 | 1.018(0.725-1.428) |
|  |  |  | GG | 122(0.455) | 125(0.460) |  | 1 (ref) |
|  |  | codominant (AG vs. GG) | AG | 232(0.655) | 227(0.645) | 0.77 | 1.047(0.769-1.427) |
|  |  |  | GG | 122(0.345) | 125(0.350) |  | 1 (ref) |
| *IL18* | *rs549908* | dominant (TT+TG vs. GG) | TT+TG | 497(0.994) | 495(0.990) | 0.725 | 1.673(0.398-7.040) |
|  |  |  | GG | 3(0.006) | 5(0.010) |  | 1 (ref) |
|  |  | recessive (TT vs. TG+GG) | TT | 393(0.786) | 373(0.746) | 0.135 | 1.251(0.932-1.677) |
|  |  |  | TG+GG | 107(0.214) | 127(0.254) |  | 1 (ref) |
|  |  | codominant (TT vs. GG) | TT | 393(0.992) | 373(0.987) | 0.496 | 1.756(0.417-7.399) |
|  |  |  | GG | 3(0.008) | 5(0.013) |  | 1 (ref) |
|  |  | codominant (TG vs. GG) | TG | 104(0.972) | 122(0.961) | 0.73 | 1.421(0.332-6.088) |
|  |  |  | GG | 3(0.028) | 5(0.039) |  | 1 (ref) |
| *IL18* | *rs360719* | dominant (AA+GA vs. GG) | AA+GA | 497(0.996) | 495(0.990) | 0.451 | 2.510(0.485-12.999) |
|  |  |  | GG | 2(0.004) | 5(0.010) |  | 1 (ref) |
|  |  | recessive (AA vs. GA+GG) | AA | 390(0.782) | 373(0.746) | 0.186 | 1.218(0.909-1.632) |
|  |  |  | GA+GG | 109(0.218) | 127(0.254) |  | 1 (ref) |
|  |  | codominant (AA vs. GG) | AA | 390(0.995) | 373(0.987) | 0.279 | 2.614(0.504-13.556) |
|  |  |  | GG | 2(0.005) | 5(0.013) |  | 1 (ref) |
|  |  | codominant (GA vs. GG) | GA | 107(0.982) | 122(0.961) | 0.456 | 2.193(0.417-11.534) |
|  |  |  | GG | 2(0.018) | 5(0.039) |  | 1 (ref) |
| *IL18* | *rs1946518* | dominant (GG+TG vs. TT) | GG+TG | 394(0.788) | 371(0.742) | 0.086 | 1.292(0.964-1.733) |
|  |  |  | TT | 106(0.212) | 129(0.258) |  | 1 (ref) |
|  |  | recessive (GG vs. TG+TT) | GG | 136(0.272) | 124(0.243) | 0.294 | 1.163(0.877-1.543) |
|  |  |  | TG+TT | 364(0.728) | 386(0.757) |  | 1 (ref) |
|  |  | codominant (GG vs. TT) | GG | 136(0.562) | 124(0.490) | 0.109 | 1.335(0.937-1.902) |
|  |  |  | TT | 106(0.438) | 129(0.510) |  | 1 (ref) |
|  |  | codominant (TG vs. TT) | TG | 258(0.709) | 247(0.657) | 0.13 | 1.271(0.932-1.734) |
|  |  |  | TT | 106(0.291) | 129(0.343) |  | 1 (ref) |
| *GSDMD* | *rs11551202* | dominant (AA+GA vs. GG) | AA+GA | 119(0.238) | 114(0.228) | 0.965 | 1.060(0.791-1.422) |
|  |  |  | GG | 380(0.762) | 386(0.772) |  | 1 (ref) |
|  |  | recessive (AA vs. GA+GG) | AA | 10(0.020) | 6(0.012) | 0.311 | 1.684(0.607-4.668) |
|  |  |  | GA+GG | 489(0.980) | 494(0.988) |  | 1 (ref) |
|  |  | codominant (AA vs. GG) | AA | 10(0.026) | 6(0.015) | 0.307 | 1.693(0.609-4.704) |
|  |  |  | GG | 380(0.974) | 386(0.985) |  | 1 (ref) |
|  |  | codominant (GA vs. GG) | GA | 109(0.223) | 108(0.219) | 0.871 | 1.025(0.759-1.386) |
|  |  |  | GG | 380(0.777) | 386(0.781) |  | 1 (ref) |
| *GSDMD* | *rs1545536* | dominant (CC+TC vs. TT) | CC+TC | 408(0.818) | 398(0.796) | 0.386 | 1.149(0.839-1.574) |
|  |  |  | TT | 91(0.182) | 102(0.204) |  | 1 (ref) |
|  |  | recessive (CC vs. TC+TT) | CC | 153(0.307) | 149(0.298) | 0.767 | 1.042(0.795-1.365) |
|  |  |  | TC+TT | 346(0.693) | 351(0.702) |  | 1 (ref) |
|  |  | codominant (CC vs. TT) | CC | 153(0.627) | 149(0.594) | 0.446 | 1.151(0.802-1.652) |
|  |  |  | TT | 91(0.373) | 102(0.406) |  | 1 (ref) |
|  |  | codominant (TC vs. TT) | TC | 255(0.737) | 249(0.709) | 0.416 | 1.148(0.823-1.600) |
|  |  |  | TT | 91(0.263) | 102(0.291) |  | 1 (ref) |
| *GSDMD* | *rs7834318* | dominant (AA+AC vs. CC) | AA+AC | 410(0.820) | 415(0.830) | 0.677 | 0.933(0.673-1.293) |
|  |  |  | CC | 90(0.180) | 85(0.170) |  | 1 (ref) |
|  |  | recessive (AA vs. AC+CC) | AA | 165(0.330) | 148(0.296) | 0.246 | 1.171(0.896-1.531) |
|  |  |  | AC+CC | 335(0.670) | 352(0.704) |  | 1 (ref) |
|  |  | codominant (AA vs. CC) | AA | 165(0.647) | 148(0.635) | 0.785 | 1.053(0.727-1.525) |
|  |  |  | CC | 90(0.353) | 85(0.365) |  | 1 (ref) |
|  |  | codominant (AC vs. CC) | AC | 245(0.731) | 267(0.759) | 0.414 | 0.867(0.615-1.222) |
|  |  |  | CC | 90(0.269) | 85(0.241) |  | 1 (ref) |
| *NLRP3* | *rs10754558* | dominant (CC+GC vs. GG) | CC+GC | 388(0.776) | 393(0.786) | 0.702 | 0.943(0.699-1.273) |
|  |  |  | GG | 112(0.224) | 107(0.214) |  | 1 (ref) |
|  |  | recessive (CC vs. GC+GG) | CC | 159(0.318) | 142(0.284) | 0.241 | 1.176(0.897-1.541) |
|  |  |  | GC+GG | 341(0.682) | 358(0.716) |  | 1 (ref) |
|  |  | codominant (CC vs. GG) | CC | 159(0.587) | 142(0.570) | 0.705 | 1.070(0.755-1.516) |
|  |  |  | GG | 112(0.413) | 107(0.430) |  | 1 (ref) |
|  |  | codominant (GC vs. GG) | GC | 229(0.672) | 251(0.701) | 0.4 | 0.872(0.633-1.200) |
|  |  |  | GG | 112(0.328) | 107(0.299) |  | 1 (ref) |
| *NLRP3* | *rs4612666* | dominant (TT+TC vs. CC) | TT+TC | 360(0.720) | 330(0.660) | **0.040** | 1.325(1.012-1.733) |
|  |  |  | CC | 140(0.280) | 170(0.340) |  | 1 (ref) |
|  |  | recessive (TT vs. TC+CC) | TT | 87(0.174) | 91(0.182) | 0.741 | 0.947(0.685-1.309) |
|  |  |  | TC+CC | 413(0.826) | 409(0.818) |  | 1 (ref) |
|  |  | codominant (TT vs. CC) | TT | 87(0.383) | 91(0.349) | 0.428 | 1.161(0.802-1.680) |
|  |  |  | CC | 140(0.617) | 170(0.651) |  | 1 (ref) |
|  |  | codominant (TC vs. CC) | TC | 273(0.661) | 239(0.584) | **0.023** | 1.387(1.045-1.841) |
|  |  |  | CC | 140(0.339) | 170(0.416) |  | 1 (ref) |
| *NLRP3* | *rs3806265* | dominant (CC+TC vs. TT) | CC+TC | 365(0.730) | 350(0.700) | 0.293 | 1.159(0.880-1.525) |
|  |  |  | TT | 135(0.270) | 150(0.300) |  | 1 (ref) |
|  |  | recessive (CC vs. TC+TT) | CC | 100(0.200) | 109(0.218) | 0.484 | 0.897(0.661-1.217) |
|  |  |  | TC+TT | 400(0.800) | 391(0.782) |  | 1 (ref) |
|  |  | codominant (CC vs. TT) | CC | 100(0.426) | 109(0.421) | 0.916 | 1.019(0.713-1.457) |
|  |  |  | TT | 135(0.574) | 150(0.579) |  | 1 (ref) |
|  |  | codominant (TC vs. TT) | TC | 265(0.663) | 241(0.616) | 0.177 | 1.222(0.914-1.634) |
|  |  |  | TT | 135(0.338) | 150(0.384) |  | 1 (ref) |
| *NLRP3* | *rs1539019* | dominant (AA+AC vs. CC) | AA+AC | 352(0.704) | 346(0.692) | 0.679 | 1.059(0.808-1.387) |
|  |  |  | CC | 148(0.296) | 154(0.308) |  | 1 (ref) |
|  |  | recessive (AA vs. AC+CC) | AA | 106(0.212) | 92(0.184) | 0.267 | 1.193(0.874-1.629) |
|  |  |  | AC+CC | 394(0.788) | 408(0.816) |  | 1 (ref) |
|  |  | codominant (AA vs. CC) | AA | 106(0.417) | 92(0.374) | 0.322 | 1.199(0.837-1.717) |
|  |  |  | CC | 148(0.583) | 154(0.626) |  | 1 (ref) |
|  |  | codominant (AC vs. CC) | AC | 246(0.624) | 254(0.623) | 0.958 | 1.008(0.757-1.341) |
|  |  |  | CC | 148(0.376) | 154(0.377) |  | 1 (ref) |
| *IL1B* | *rs4848306* | dominant (AA+GA vs. GG) | AA+GA | 376(0.754) | 363(0.726) | 0.322 | 1.154(0.869-1.531) |
|  |  |  | GG | 123(0.246) | 137(0.274) |  | 1 (ref) |
|  |  | recessive (AA vs. GA+GG) | AA | 120(0.240) | 121(0.242) | 0.955 | 0.992(0.742-1.325) |
|  |  |  | GA+GG | 379(0.760) | 379(0.758) |  | 1 (ref) |
|  |  | codominant (AA vs. GG) | AA | 120(0.494) | 121(0.469) | 0.578 | 1.105(0.778-1.569) |
|  |  |  | GG | 123(0.506) | 137(0.531) |  | 1 (ref) |
|  |  | codominant (GA vs. GG) | GA | 256(0.675) | 242(0.639) | 0.284 | 1.178(0.873-1.591) |
|  |  |  | GG | 123(0.325) | 137(0.361) |  | 1 (ref) |
| *IL1B* | *rs3136558* | dominant (AA+GA vs. GG) | AA+GA | 433(0.868) | 421(0.842) | 0.248 | 1.231(0.865-1.753) |
|  |  |  | GG | 66(0.132) | 79(0.158) |  | 1 (ref) |
|  |  | recessive (AA vs. GA+GG) | AA | 184(0.369) | 173(0.346) | 0.453 | 1.104(0.852-1.430) |
|  |  |  | GA+GG | 315(0.631) | 327(0.654) |  | 1 (ref) |
|  |  | codominant (AA vs. GG) | AA | 184(0.736) | 173(0.687) | 0.221 | 1.273(0.864-1.875) |
|  |  |  | GG | 66(0.264) | 79(0.313) |  | 1 (ref) |
|  |  | codominant (GA vs. GG) | GA | 249(0.790) | 248(0.758) | 0.331 | 1.202(0.829-1.742) |
|  |  |  | GG | 66(0.210) | 79(0.242) |  | 1 (ref) |
| *IL1B* | *rs2853550* | dominant (GG+GA vs. AA) | GG+GA | 494(0.988) | 498(0.996) | 0.287 | 0.331(0.066-1.646) |
|  |  |  | AA | 6(0.012) | 2(0.004) |  | 1 (ref) |
|  |  | recessive (GG vs. GA+AA) | GG | 412(0.824) | 399(0.798) | 0.249 | 1.185(0.863-1.628) |
|  |  |  | GA+AA | 88(0.176) | 101(0.202) |  | 1 (ref) |
|  |  | codominant (GG vs. AA) | GG | 412(0.986) | 399(0.995) | 0.288 | 0.344(0.069-1.715) |
|  |  |  | AA | 6(0.014) | 2(0.005) |  | 1 (ref) |
|  |  | codominant (GA vs. AA) | GA | 82(0.932) | 99(0.980) | 0.148 | 0.276(0.054-1.405) |
|  |  |  | AA | 6(0.068) | 2(0.020) |  | 1 (ref) |
| *IL1B* | *rs16944* | dominant (GG+GA vs. AA) | GG+GA | 397(0.794) | 376(0.752) | 0.113 | 1.271(0.945-1.711) |
|  |  |  | AA | 103(0.206) | 124(0.248) |  | 1 (ref) |
|  |  | recessive (GG vs. GA+AA) | GG | 135(0.270) | 135(0.270) | 1.000 | 1.000(0.756-1.322) |
|  |  |  | GA+AA | 365(0.730) | 365(0.730) |  | 1 (ref) |
|  |  | codominant (GG vs. AA) | GG | 135(0.567) | 135(0.521) | 0.304 | 1.204(0.845-1.715) |
|  |  |  | AA | 103(0.433) | 124(0.479) |  | 1 (ref) |
|  |  | codominant (GA vs. AA) | GA | 262(0.718) | 241(0.660) | 0.093 | 1.309(0.956-1.792) |
|  |  |  | AA | 103(0.282) | 124(0.340) |  | 1 (ref) |
| *IL1B* | *rs1143623* | dominant (CC+GC vs. GG) | CC+GC | 425(0.850) | 409(0.818) | 0.174 | 1.261(0.902-1.762) |
|  |  |  | GG | 75(0.150) | 91(0.182) |  | 1 (ref) |
|  |  | recessive (CC vs. GC+GG) | CC | 180(0.360) | 169(0.338) | 0.466 | 1.102(0.849-1.429) |
|  |  |  | GC+GG | 320(0.640) | 331(0.662) |  | 1 (ref) |
|  |  | codominant (CC vs. GG) | CC | 75(0.294) | 91(0.350) | 0.175 | 0.774(0.534-1.121) |
|  |  |  | GG | 180(0.706) | 169(0.650) |  | 1 (ref) |
|  |  | codominant (GC vs. GG) | GC | 245(0.766) | 240(0.725) | 0.235 | 1.239(0.870-1.764) |
|  |  |  | GG | 75(0.234) | 91(0.275) |  | 1 (ref) |
| *MAVS* | *rs7262903* | dominant (AA+AC vs. CC) | AA+AC | 116(0.232) | 98(0.196) | 0.165 | 1.239(0.915-1.678) |
|  |  |  | CC | 384(0.768) | 402(0.804) |  | 1 (ref) |
|  |  | recessive (AA vs. AC+CC) | AA | 7(0.014) | 6(0.012) | 0.780 | 1.169(0.390-3.503) |
|  |  |  | AC+CC | 493(0.986) | 494(0.988) |  | 1 (ref) |
|  |  | codominant (AA vs. CC) | AA | 7(0.018) | 6(0.015) | 0.721 | 1.221(0.407-3.667) |
|  |  |  | CC | 384(0.982) | 402(0.985) |  | 1 (ref) |
|  |  | codominant (AC vs. CC) | AC | 109(0.221) | 92(0.186) | 0.174 | 1.240(0.909-1.692) |
|  |  |  | CC | 384(0.779) | 402(0.814) |  | 1 (ref) |
| *MAVS* | *rs17857295* | dominant (CC+GC vs. GG) | CC+GC | 389(0.780) | 363(0.726) | 0.050 | 1.335(1.000-1.782) |
|  |  |  | GG | 110(0.220) | 137(0.274) |  | 1 (ref) |
|  |  | recessive (CC vs. GC+GG) | CC | 123(0.246) | 125(0.250) | 0.898 | 0.981(0.736-1.308) |
|  |  |  | GC+GG | 376(0.754) | 375(0.750) |  | 1 (ref) |
|  |  | codominant (CC vs. GG) | CC | 123(0.528) | 125(0.477) | 0.259 | 1.226(0.861-1.745) |
|  |  |  | GG | 110(0.472) | 137(0.523) |  | 1 (ref) |
|  |  | codominant (GC vs. GG) | GC | 266(0.707) | 238(0.635) | **0.034** | 1.392(1.025-1.890) |
|  |  |  | GG | 110(0.293) | 137(0.365) |  | 1 (ref) |
| *MAVS* | *rs6084497* | dominant (TT+TC vs. CC) | TT+TC | 311(0.622) | 280(0.560) | **0.046** | 1.293(1.004-1.665) |
|  |  |  | CC | 189(0.378) | 220(0.440) |  | 1 (ref) |
|  |  | recessive (TT vs. TC+CC) | TT | 71(0.142) | 61(0.122) | 0.350 | 1.191(0.825-1.719) |
|  |  |  | TC+CC | 429(0.858) | 439(0.878) |  | 1 (ref) |
|  |  | codominant (TT vs. CC) | TT | 71(0.273) | 61(0.217) | 0.130 | 1.355(0.914-2.008) |
|  |  |  | CC | 189(0.727) | 220(0.783) |  | 1 (ref) |
|  |  | codominant (TC vs. CC) | TC | 240(0.559) | 219(0.499) | 0.074 | 1.276(0.977-1.666) |
|  |  |  | CC | 189(0.441) | 220(0.501) |  | 1 (ref) |
| *MAVS* | *rs16989000* | dominant (CC+CA vs. AA) | CC+CA | 335(0.670) | 304(0.608) | **0.041** | 1.309(1.011-1.696) |
|  |  |  | AA | 165(0.330) | 196(0.392) |  | 1 (ref) |
|  |  | recessive (CC vs. CA+AA) | CC | 82(0.164) | 70(0.140) | 0.291 | 1.205(0.852-1.704) |
|  |  |  | CA+AA | 418(0.836) | 430(0.860) |  | 1 (ref) |
|  |  | codominant (CC vs. AA) | CC | 82(0.332) | 70(0.263) | 0.088 | 1.392(0.951-2.035) |
|  |  |  | AA | 165(0.668) | 196(0.737) |  | 1 (ref) |
|  |  | codominant (CA vs. AA) | CA | 254(0.606) | 234(0.544) | 0.068 | 1.289(0.982-1.694) |
|  |  |  | AA | 165(0.394) | 196(0.456) |  | 1 (ref) |
| *MAVS* | *rs6515831* | dominant (TT+TC vs. CC) | TT+TC | 479(0.958) | 468(0.936) | 0.121 | 1.560(0.886-2.744) |
|  |  |  | CC | 21(0.042) | 32(0.064) |  | 1 (ref) |
|  |  | recessive (TT vs. TC+CC) | TT | 286(0.572) | 289(0.578) | 0.848 | 0.976(0.759-1.254) |
|  |  |  | TC+CC | 214(0.428) | 211(0.422) |  | 1 (ref) |
|  |  | codominant (TT vs. CC) | TT | 286(0.932) | 289(0.900) | 0.159 | 1.508(0.849-2.678) |
|  |  |  | CC | 21(0.068) | 32(0.100) |  | 1 (ref) |
|  |  | codominant (TC vs. CC) | TC | 193(0.902) | 179(0.848) | 0.095 | 1.643(0.914-2.954) |
|  |  |  | CC | 21(0.098) | 32(0.152) |  | 1 (ref) |
| *MAVS* | *rs57173648* | dominant (TT+TC vs. CC) | TT+TC | 62(0.124) | 63(0.126) | 0.924 | 0.982(0.675-1.428) |
|  |  |  | CC | 438(0.876) | 437(0.874) |  | 1 (ref) |
|  |  | recessive (TT vs. TC+CC) | TT | 2(0.004) | 1(0.002) | 1.000 | 2.004(0.181-22.172) |
|  |  |  | TC+CC | 498(0.996) | 499(0.998) |  | 1 (ref) |
|  |  | codominant (TT vs. CC) | TT | 2(0.005) | 1(0.002) | 1.000 | 1.995(0.180-22.087) |
|  |  |  | CC | 438(0.995) | 437(0.998) |  | 1 (ref) |
|  |  | codominant (TC vs. CC) | TC | 60(0.12) | 62(0.124) | 0.856 | 0.966(0.661-1.410) |
|  |  |  | CC | 438(0.88) | 437(0.876) |  | 1 (ref) |
| *MAVS* | *rs867335* | dominant (AA+AT vs. TT) | AA+AT | 233(0.467) | 212(0.424) | 0.898 | 1.036(0.607-1.768) |
|  |  |  | TT | 266(0.533) | 288(0.576) |  | 1 (ref) |
|  |  | recessive (AA vs. AT+TT) | AA | 28(0.056) | 29(0.058) | 0.172 | 0.840(0.655-1.079) |
|  |  |  | AT+TT | 471(0.944) | 471(0.942) |  | 1 (ref) |
|  |  | codominant (AA vs. TT) | AA | 28(0.095) | 29(0.091) | 0.873 | 1.045(0.606-1.804) |
|  |  |  | TT | 266(0.905) | 288(0.909) |  | 1 (ref) |
|  |  | codominant (AT vs. TT) | AT | 205(0.435) | 183(0.389) | 0.145 | 1.213(0.935-1.573) |
|  |  |  | TT | 266(0.565) | 288(0.611) |  | 1 (ref) |
| *JAK1* | *rs7531799* | dominant (TT+TC vs. CC) | TT+TC | 358(0.716) | 355(0.710) | 0.834 | 1.030(0.783-1.354) |
|  |  |  | CC | 142(0.284) | 145(0.290) |  | 1 (ref) |
|  |  | recessive (TT vs. TC+CC) | TT | 115(0.230) | 106(0.212) | 0.493 | 1.110(0.823-1.497) |
|  |  |  | TC+CC | 385(0.770) | 394(0.788) |  | 1 (ref) |
|  |  | codominant (TT vs. CC) | TT | 115(0.447) | 106(0.422) | 0.567 | 1.108(0.780-1.574) |
|  |  |  | CC | 142(0.553) | 145(0.578) |  | 1 (ref) |
|  |  | codominant (TC vs. CC) | TC | 243(0.631) | 249(0.632) | 0.981 | 0.997(0.745-1.333) |
|  |  |  | CC | 142(0.369) | 145(0.368) |  | 1 (ref) |
| *JAK1* | *rs4244165* | dominant (TT+TG vs. GG) | TT+TG | 287(0.575) | 256(0.512) | **0.045** | 1.290(1.005-1.656) |
|  |  |  | GG | 212(0.425) | 244(0.488) |  | 1 (ref) |
|  |  | recessive (TT vs. TG+GG) | TT | 52(0.104) | 59(0.118) | 0.488 | 0.870(0.586-1.291) |
|  |  |  | TG+GG | 447(0.896) | 441(0.882) |  | 1 (ref) |
|  |  | codominant (TT vs. GG) | TT | 52(0.197) | 59(0.195) | 0.946 | 1.014(0.669-1.537) |
|  |  |  | GG | 212(0.803) | 244(0.805) |  | 1 (ref) |
|  |  | codominant (TG vs. GG) | TG | 235(0.526) | 197(0.447) | **0.019** | 1.373(1.054-1.788) |
|  |  |  | GG | 212(0.474) | 244(0.553) |  | 1 (ref) |
| *JAK1* | *rs1039125* | dominant (TT+TC vs. CC) | TT+TC | 414(0.828) | 407(0.814) | 0.564 | 1.100(0.796-1.520) |
|  |  |  | CC | 86(0.172) | 93(0.186) |  | 1 (ref) |
|  |  | recessive (TT vs. TC+CC) | TT | 173(0.346) | 162(0.324) | 0.461 | 1.104(0.849-1.436) |
|  |  |  | TC+CC | 327(0.654) | 338(0.676) |  | 1 (ref) |
|  |  | codominant (TT vs. CC) | TT | 173(0.668) | 162(0.635) | 0.437 | 1.155(0.803-1.660) |
|  |  |  | CC | 86(0.332) | 93(0.365) |  | 1 (ref) |
|  |  | codominant (TC vs. CC) | TC | 241(0.737) | 245(0.725) | 0.724 | 1.064(0.755-1.499) |
|  |  |  | CC | 86(0.263) | 93(0.275) |  | 1 (ref) |
| *JAK1* | *rs56818621* | dominant (CC+GC vs. GG) | CC+GC | 423(0.848) | 418(0.836) | 0.612 | 1.092(0.777-1.534) |
|  |  |  | GG | 76(0.152) | 82(0.164) |  | 1 (ref) |
|  |  | recessive (CC vs. GC+GG) | CC | 198(0.397) | 180(0.360) | 0.231 | 1.169(0.905-1.511) |
|  |  |  | GC+GG | 301(0.603) | 320(0.640) |  | 1 (ref) |
|  |  | codominant (CC vs. GG) | CC | 198(0.723) | 180(0.687) | 0.366 | 1.187(0.818-1.721) |
|  |  |  | GG | 76(0.277) | 82(0.313) |  | 1 (ref) |
|  |  | codominant (GC vs. GG) | GC | 225(0.748) | 238(0.744) | 0.914 | 1.020(0.711-1.464) |
|  |  |  | GG | 76(0.252) | 82(0.256) |  | 1 (ref) |
| *JAK1* | *rs11579758* | dominant (AA+GA vs. GG) | AA+GA | 297(0.594) | 279(0.558) | 0.249 | 1.159(0.902-1.490) |
|  |  |  | GG | 203(0.406) | 221(0.442) |  | 1 (ref) |
|  |  | recessive (AA vs. GA+GG) | AA | 61(0.122) | 57(0.114) | 0.695 | 1.080(0.375-1.586) |
|  |  |  | GA+GG | 439(0.878) | 443(0.886) |  | 1 (ref) |
|  |  | codominant (AA vs. GG) | AA | 61(0.231) | 57(0.205) | 0.463 | 1.165(0.775-1.752) |
|  |  |  | GG | 203(0.769) | 221(0.795) |  | 1 (ref) |
|  |  | codominant (GA vs. GG) | GA | 236(0.538) | 222(0.501) | 0.279 | 1.157(0.888-1.508) |
|  |  |  | GG | 203(0.462) | 221(0.499) |  | 1 (ref) |
| *JAK1* | *rs567354* | dominant (AA+GA vs. GG) | AA+GA | 333(0.666) | 308(0.616) | 0.099 | 1.243(0.960-1.610) |
|  |  |  | GG | 167(0.334) | 192(0.384) |  | 1 (ref) |
|  |  | recessive (AA vs. GA+GG) | AA | 73(0.146) | 80(0.160) | 0.539 | 0.898(0.636-1.267) |
|  |  |  | GA+GG | 427(0.854) | 420(0.840) |  | 1 (ref) |
|  |  | codominant (AA vs. GG) | AA | 73(0.304) | 80(0.294) | 0.804 | 1.049(0.718-1.533) |
|  |  |  | GG | 167(0.696) | 192(0.706) |  | 1 (ref) |
|  |  | codominant (GA vs. GG) | GA | 260(0.609) | 228(0.543) | 0.052 | 1.311(0.998-1.723) |
|  |  |  | GG | 167(0.391) | 192(0.457) |  | 1 (ref) |
| *JAK1* | *rs490178* | dominant (GG+GA vs. AA) | GG+GA | 242(0.485) | 222(0.444) | 0.194 | 1.179(0.919-1.512) |
|  |  |  | AA | 257(0.515) | 278(0.556) |  | 1 (ref) |
|  |  | recessive (GG vs. GA+AA) | GG | 32(0.064) | 35(0.070) | 0.711 | 0.910(0.554-1.495) |
|  |  |  | GA+AA | 467(0.936) | 465(0.930) |  | 1 (ref) |
|  |  | codominant (GG vs. AA) | GG | 32(0.111) | 35(0.112) | 0.966 | 0.989(0.595-1.644) |
|  |  |  | AA | 257(0.889) | 278(0.888) |  | 1 (ref) |
|  |  | codominant (GA vs. AA) | GA | 210(0.450) | 187(0.402) | 0.142 | 1.215(0.937-1.575) |
|  |  |  | AA | 257(0.550) | 278(0.598) |  | 1 (ref) |
| *JAK1* | *rs705509* | dominant (GG+GA vs. AA) | GG+GA | 406(0.814) | 408(0.816) | 0.923 | 0.984(0.715-1.355) |
|  |  |  | AA | 93(0.186) | 92(0.184) |  | 1 (ref) |
|  |  | recessive (GG vs. GA+AA) | GG | 162(0.325) | 144(0.288) | 0.209 | 1.188(0.908-1.556) |
|  |  |  | GA+AA | 337(0.675) | 356(0.712) |  | 1 (ref) |
|  |  | codominant (GG vs. AA) | GG | 162(0.635) | 144(0.610) | 0.566 | 1.113(0.772-1.604) |
|  |  |  | AA | 93(0.365) | 92(0.390) |  | 1 (ref) |
|  |  | codominant (GA vs. AA) | GA | 244(0.724) | 264(0.742) | 0.602 | 0.914(0.653-1.280) |
|  |  |  | AA | 93(0.276) | 92(0.258) |  | 1 (ref) |
| *JAK1* | *rs489500* | dominant (CC+GC vs. GG) | CC+GC | 446(0.894) | 444(0.888) | 0.769 | 1.061(0.713-1.580) |
|  |  |  | GG | 53(0.106) | 56(0.112) |  | 1 (ref) |
|  |  | recessive (CC vs. GC+GG) | CC | 228(0.457) | 227(0.454) | 0.926 | 1.012(0.789-1.298) |
|  |  |  | GC+GG | 271(0.543) | 273(0.546) |  | 1 (ref) |
|  |  | codominant (CC vs. GG) | CC | 228(0.811) | 227(0.802) | 0.780 | 1.061(0.699-1.612) |
|  |  |  | GG | 53(0.189) | 56(0.198) |  | 1 (ref) |
|  |  | codominant (GC vs. GG) | GC | 218(0.804) | 217(0.795) | 0.781 | 1.061(0.697-1.616) |
|  |  |  | GG | 53(0.196) | 56(0.205) |  | 1 (ref) |
| *JAK1* | *rs310241* | dominant (AA+GA vs. GG) | AA+GA | 462(0.926) | 456(0.912) | 0.423 | 1.205(0.764-1.901) |
|  |  |  | GG | 37(0.074) | 44(0.088) |  | 1 (ref) |
|  |  | recessive (AA vs. GA+GG) | AA | 274(0.549) | 249(0.498) | 0.106 | 1.228(0.957-1.574) |
|  |  |  | GA+GG | 225(0.451) | 251(0.502) |  | 1 (ref) |
|  |  | codominant (AA vs. GG) | AA | 274(0.881) | 249(0.850) | 0.261 | 1.309(0.818-2.093) |
|  |  |  | GG | 37(0.119) | 44(0.150) |  | 1 (ref) |
|  |  | codominant (GA vs. GG) | GA | 188(0.836) | 207(0.825) | 0.753 | 1.080(0.669-1.745) |
|  |  |  | GG | 37(0.164) | 44(0.175) |  | 1 (ref) |
| ^a^Results are shown as n (frequency). | | | | | | | |
| Bold type indicates statistical significance (*p* < 0.05). | | | | | | | |
