# Supplementary figures and images for: Genetic Variants in the NOD-like Receptor Signaling Pathway Are Associated with HIV-1/AIDS in a Northern Chinese Population
Source: Int J Mol Sci. 2025 Apr 8;26(8):3484. doi: 10.3390/ijms26083484 (PMC12026778; doi:10.3390/ijms26083484)

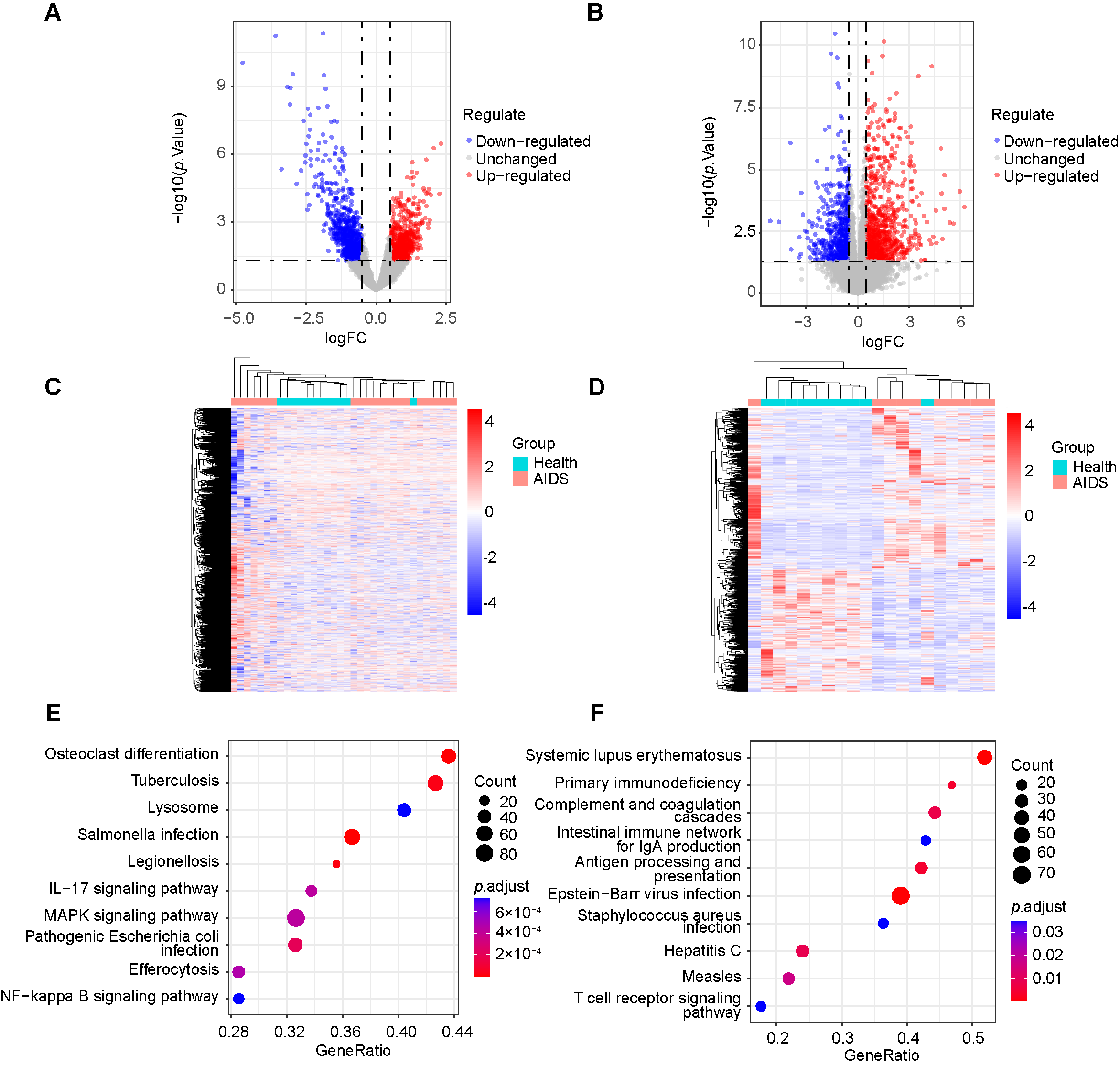

Supplement: Supplementary file 1 [file ijms-26-03484-s001.zip › Figure_S1.tif]

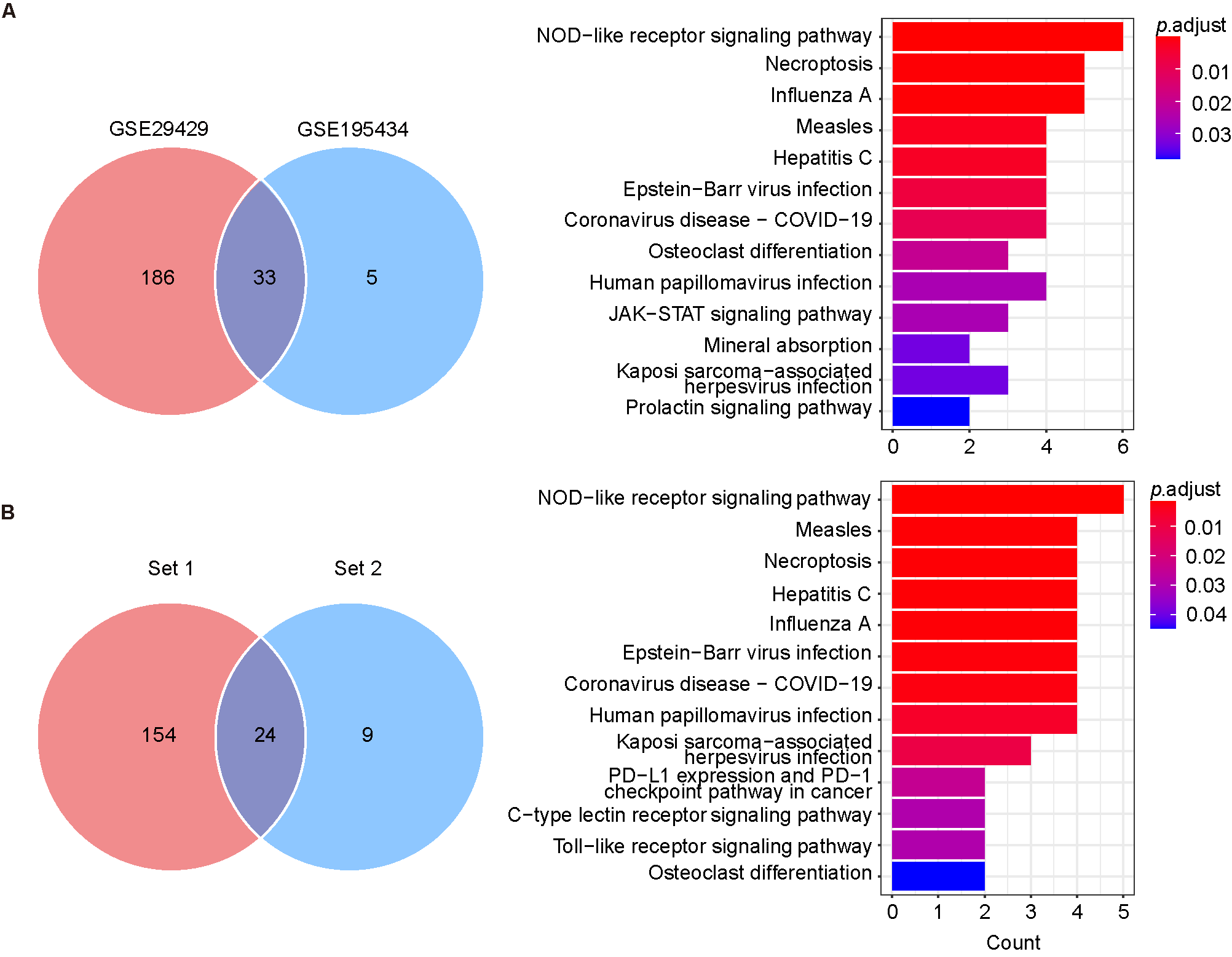

Supplement: Supplementary file 1 [file ijms-26-03484-s001.zip › Figure_S2.tif]

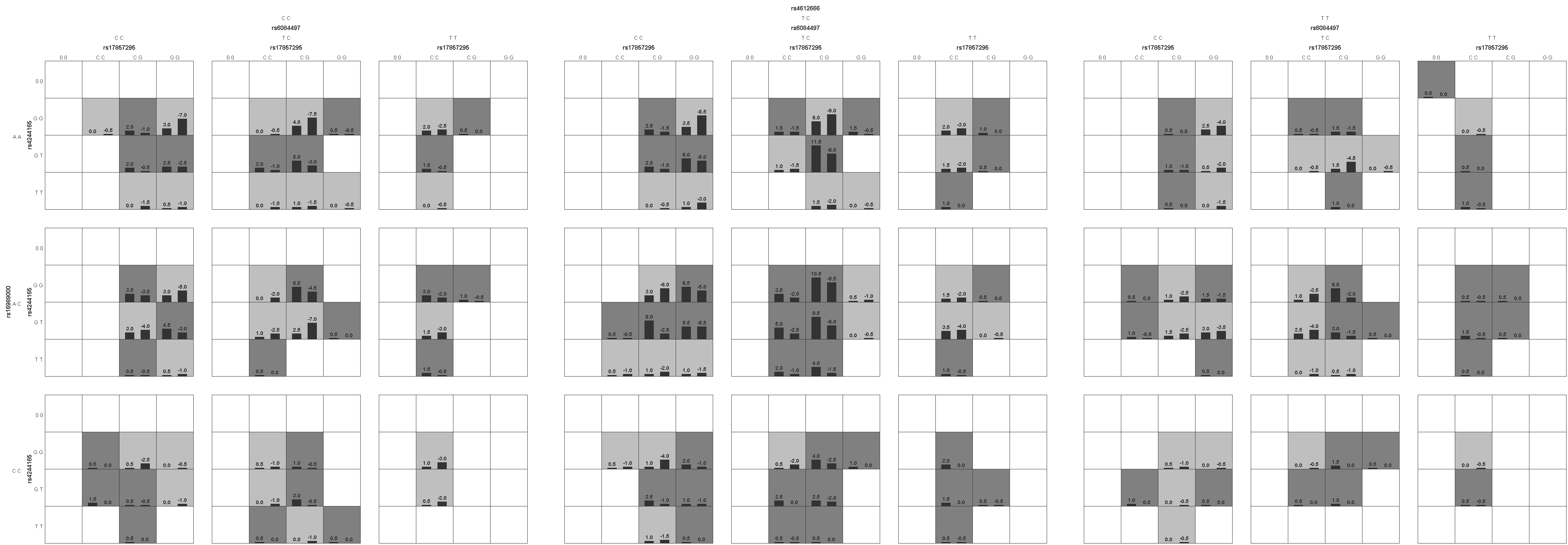

Supplement: Supplementary file 1 [file ijms-26-03484-s001.zip › Figure_S3.png]

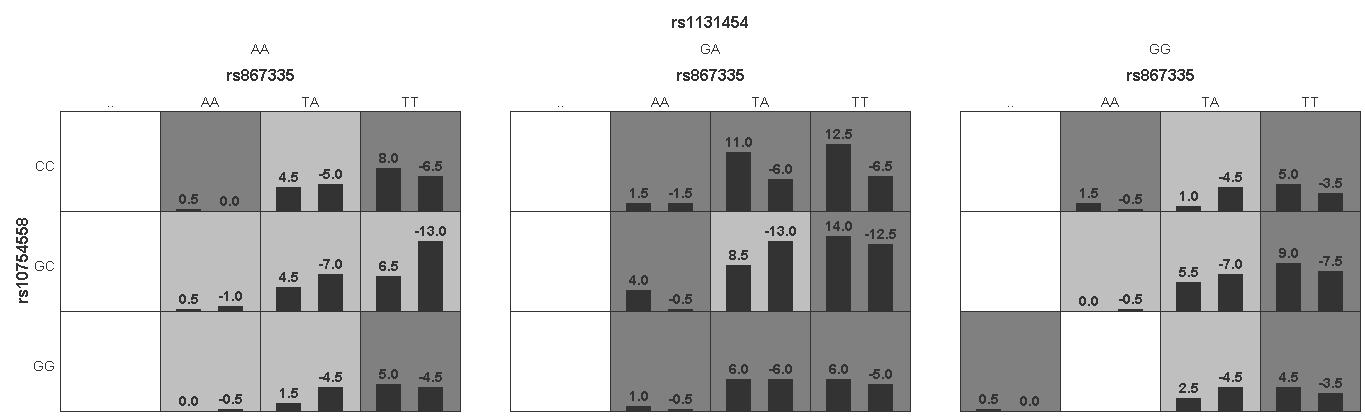

Supplement: Supplementary file 1 [file ijms-26-03484-s001.zip › Figure_S4.jpg]
